# Brazilian montane rainforest expansion induced by Heinrich Stadial 1 event

**DOI:** 10.1038/s41598-019-53036-1

**Published:** 2019-11-29

**Authors:** Jorge L. D. Pinaya, Francisco W. Cruz, Gregório C. T. Ceccantini, Pedro L. P. Corrêa, Nigel Pitman, Felipe Vemado, Maria del Carmen S. Lopez, Augusto J. Pereira Filho, Carlos H. Grohmann, Cristiano M. Chiessi, Nicolás M. Stríkis, Ingrid Horák-Terra, Walter H. L. Pinaya, Vanda B. de Medeiros, Rudney de A. Santos, Thomas K. Akabane, Maicon A. Silva, Rachid Cheddadi, Mark Bush, Alexandra-Jane Henrot, Louis François, Alain Hambuckers, Frédéric Boyer, Matthieu Carré, Eric Coissac, Francesco Ficetola, Kangyou Huang, Anne-Marie Lézine, Majda Nourelbait, Ali Rhoujjati, Pierre Taberlet, Fausto Sarmiento, Daniel Abel-Schaad, Francisca Alba-Sánchez, Zhuo Zheng, Paulo E. De Oliveira

**Affiliations:** 1 Politechnical School, University of São Paulo, São Paulo, SP Brazil; 2 Institute of Geosciences, University of São Paulo, São Paulo, SP Brazil; 3 Science Action, The Field Museum of Natural History, Chicago, Illinois US; 4 Institute of Biosciences, University of São Paulo, São Paulo, SP Brazil; 5 Institute of Astronomy and Geophysics and Atmospheric Sciences, University of São Paulo, São Paulo, SP Brazil; 6 Institute of Energy and Environment, University of São Paulo, São Paulo, SP Brazil; 7 School of Arts, Sciences and Humanities, University of São Paulo, São Paulo, SP Brazil; 8 Federal Fluminense University, Niteroi, RJ Brazil; 9 Institute of Agricultural Sciences, Federal University of Jequitinhonha and Mucuri Valleys, Unaí, MG Brazil; 10 Center of Mathematics, Computation, and Cognition, Federal University of ABC, Santo André, SP Brazil; 11 ISEM, Université de Montpellier, Centre National de la Recherche Scientifique, IRD, EPHE, Montpellier, France; 12 Department of Biological Sciences, Florida Institute of Technology, Melbourne, FL US; 13 Unité de Modélisation du Climat et des Cycles Biogéochimiques, UR-SPHERES, University of Liège, Liège, Belgium; 14 Unité de Biologie du comportement, UR-SPHERES, University of Liège, Liège, Belgium; 15 Laboratoire d’Ecologie Alpine, Centre National de la Recherche Scientifique, Université Grenoble Alpes, Grenoble, France; 16 LOCEAN Laboratory, Sorbonne Universités (UPMC), CNRS, IRD, MNHN, Paris, France; 17 School of Earth Science and Geological Engineering, Sun Yat-Sen University, Guangzhou, China; 18 Laboratoire Géoressources, Unité de Recherche Associée CNRST (URAC 42), Faculté des Sciences et Techniques, Université Cadi Ayyad, Marrakech, Morocco; 19 Neotropical Montology Collaboratory, Department of Geography, University of Georgia, Athens, GA US; 20 Departamento de Botánica, Facultad de Ciencias, Universidad de Granada, Granada, Andalucia Spain

**Keywords:** Biogeography, Climate-change ecology, Palaeoecology, Palaeoclimate, Palaeoecology

## Abstract

The origin of modern disjunct plant distributions in the Brazilian Highlands with strong floristic affinities to distant montane rainforests of isolated mountaintops in the northeast and northern Amazonia and the Guyana Shield remains unknown. We tested the hypothesis that these unexplained biogeographical patterns reflect former ecosystem rearrangements sustained by widespread plant migrations possibly due to climatic patterns that are very dissimilar from present-day conditions. To address this issue, we mapped the presence of the montane arboreal taxa *Araucaria, Podocarpus, Drimys, Hedyosmum, Ilex, Myrsine, Symplocos*, and *Weinmannia*, and cool-adapted plants in the families Myrtaceae, Ericaceae, and Arecaceae (palms) in 29 palynological records during Heinrich Stadial 1 Event, encompassing a latitudinal range of 30°S to 0°S. In addition, Principal Component Analysis and Species Distribution Modelling were used to represent past and modern habitat suitability for *Podocarpus* and *Araucaria*. The data reveals two long-distance patterns of plant migration connecting south/southeast to northeastern Brazil and Amazonia with a third short route extending from one of them. Their paleofloristic compositions suggest a climatic scenario of abundant rainfall and relative lower continental surface temperatures, possibly intensified by the effects of polar air incursions forming cold fronts into the Brazilian Highlands. Although these taxa are sensitive to changes in temperature, the combined pollen and speleothems proxy data indicate that this montane rainforest expansion during Heinrich Stadial 1 Event was triggered mainly by a less seasonal rainfall regime from the subtropics to the equatorial region.

## Introduction

The origin of disjunct vegetation types in mountain landscapes of southeastern and central Brazil that display a strong affinity to wet montane floras of northern South America remains unknown. Earlier hypotheses^1,2^ suggested cold and wet migration corridors possibly in the Eocene or Miocene, later affected by the Quaternary climatic change, might have allowed ancient contact between plant populations now isolated on distant mountaintops.

In this study we investigate the impact on tropical montane vegetation of an enhanced South American Summer Monsoon (SASM) regime between 18.1 and 14.7 kcal yr BP in synchrony with glacial episodic iceberg discharge in the North Atlantic, known as Heinrich Stadial 1 (HS1), as indicated by speleothem isotope records^3–7^. Oxygen isotopes in the Botuverá cave speleothems have indicated that wet phases prevailed during the last glacial cycle in southern Brazil^8^. Additional support for this scenario comes from calcite deposits at lake margins within caves and expansion of wet forests in the HS1 of northern Bahia, currently covered by semi-arid vegetation (caatinga), suggested by abundant plant megafossils in calcareous tufas, belonging to arboreal and herbaceous taxa presently found in the Atlantic and Amazon rainforests^9^.

We hypothesize that the intensified precipitation within the area climatologically affected by the South Atlantic Convergence Zone (SACZ) and the Intertropical Convergence Zone (ITCZ) promoted conditions suitable for the establishment of north-south migration corridors for the expansion of montane forest. These possible former connections between southeast and central Brazil, from 31° to 22°S lat. and southeastern Amazonia could, therefore, explain much of the modern occurrence of disjunct humid and cold-adapted taxa in elevated areas of cerrado and semi-arid caatinga reaching 4°S, with counterparts in the tepuis of the Guyana Shield, including those in Roraima (northern Brazilian Amazonia) and in Venezuela.

## Methods

We infer vegetational and correlated climatic changes during the HS1 event by analyzing selected arboreal pollen taxa, in most cases with abundance higher than 5%, in Brazilian Late Quaternary pollen records, with morphological features that allow identification to genus level and in some cases only to family^10^, the exception of which is genus *Araucaria* Juss., represented in Brazil only by *A. angustifolia* (Bert.) O. Kuntze. The selected cold/mild and cold-humid adapted genera are *Araucaria* Juss.*, Podocarpus* L’Hér. ex Pers.*, Drimys* J.R. Forst. & G. Forst.*, Hedyosmum* Sw.*, Ilex* L.*, Myrsine* L.*, Symplocos* Jacq., and *Weinmannia* L., as well as the families Myrtaceae Juss., Ericaceae Juss. and Arecaceae Bercht. & J. Presl (*sin*. Palmae Juss., palms), chosen based on their frequent presence in glacial pollen signals of tropical America^11–14^. It is noteworthy that in southern and southeastern Brazil, *Podocarpus* is represented by two species in the highlands, *i.e. P. lambertii* Klotzsch ex Endl. and *P. sellowii* Klotzsch ex Endl. Pollen rain analyses in this area^15^ indicate that its pollen counts can be as low as 0.8% to indicate significant presence in native coastal rainforests.

These taxa are stenopalynous; although some of them may contain thousands of members, their pollen is represented only by one morphological type, and thus does not permit separation of species i.e., Myrtaceae Juss. Although palm pollen can be assigned to different genera, it frequently appears in pollen records simply as Arecaceae or Palmae.

Montane species of the families Myrtaceae, Ericaceae and Arecaceae were verified in herbarium collections of The Field Museum of Natural History and the Department of Botany of the Institute of Biological Sciences of the University of Sao Paulo and in the Flora do Brasil 2020^16^. Myrtaceae is represented by a total of 17 genera and 53 species. Ericaceae, an almost exclusive montane family of shrubs and trees comprised of 12 genera, 99 species and 27 varieties, commonly found in high altitude montane ecosystems of Brazil where they are subjected to sub-zero temperatures at certain periods of the year^2^. The palm family Arecaceae is represented on montane with subtropical humid climate by 5 genera and 19 species. A list of montane species for these three families are given in Supplementary Information.

A detailed survey of the Late Pleistocene palynological literature in Brazil reveals a total of 29 pollen profiles from continental sedimentary records containing HS1 age sediments: 1. Cambará do Sul^17^; 2. São Francisco de Assis^18^; 3. Serra da Boa Vista^19^; 4. Serra do Tabuleiro^20^; 5. Pato Branco^21^; 6. Volta Velha^22^; 7. Curucutu^23^; 8. Colônia Crater^14^; 9. Serra de Botucatu^24^; 10. Monte Verde^25^; 11. Lagoa de Itaipu^26^; 12. Morro do Itapeva^15^; 13. Lagoa dos Olhos^26,27^; 14. Salitre^13^; 15. Serra Negra^12^; 16. Brejo do Louro^28^; 17. Serra do Espinhaço^29^; 18. Buritizeiro^30^; 19. and 20. Crominia^31^; 21. Turfa de Inhumas^32^; 22. Lagoa Bonita^33^; 23. Águas Emendadas^34^; 24. Chapada dos Veadeiros^35^; 25. and 26. Serra dos Carajas^36,37^; 27. Lago Caçó^38^; 28. and 29. Lagoa da Pata^11,39^.

Four of these records are from the lowlands of equatorial regions of eastern^37^ and western Amazonia^39,40^ and one study is from Lagoa do Caçó^41^, at the easternmost Amazonian forest/savanna transition in the State of Maranhão, close to the Atlantic coast in northern Brazil. The remaining pollen records are distributed in montane forests of the Brazilian highlands, a patch of remaining humid vegetation within the modern Cerrado (Brazilian Savanna) domain of southeastern and central Brazil.

Since the physiognomy of montane forests, specially in southern and southeastern Brazil are characterized in general by an 30–40 m high emergent layer containing *Araucaria* underlain by a sub-canopy containing *Podocarpus*, we mapped their potential distribution during the late glacial phase and under current conditions. Species Distribution Model (SDM) were generated with MaxEnt^42,43^ version 3.3.3k, for the HS1 phase in the Brazilian Highlands, correlating pollen records with monthly mean convective precipitation rate (PRECC), large-scale precipitation rate (PRECL), surface (TS) and minimum surface temperature (TSMIN) from CCSM3 Trace21k dataset^44^, at a resolution finer than the 3.5° × 3.5° grid. Potential distribution maps of *Podocarpus* (20 training and 4 test samples, 0.965 average training AUC for the replicate run and 0.012 standard deviation) and *Araucaria* (9 training and 2 test samples, 0.976 average training AUC for the replicate runs and 0.006 standard deviation).

Modern Species Distribution models (SDM) for *Araucaria* (represented only by *A. angustifolia*) and *Podocarpus* (represented by *P. lambertii* and *P. sellowii*) were generated with MaxEnt^42,43^ version 3.3.3k with bootstrap resampling of 20 replicates, using 19 bioclimatic variables, obtained from worldclim version 2.0, at a resolution finer than the 1 km × 1 km grid, and modern occurrence points of SpeciesLink and SiBBr/GBIF: *Araucaria angustifolia* (124 training and 13 test samples, 0.987 average training AUC for the replicate runs and 0.001 standard deviation), *Podocarpus lambertii* (157 training and 17 test samples, 0.984 average training AUC for the replicate runs and 0.001standard deviation) and *Podocarpus sellowii* (124 training and 13 test samples, 0.987 average training AUC for the replicate run and 0.001standard deviation).

**Figure 1:**
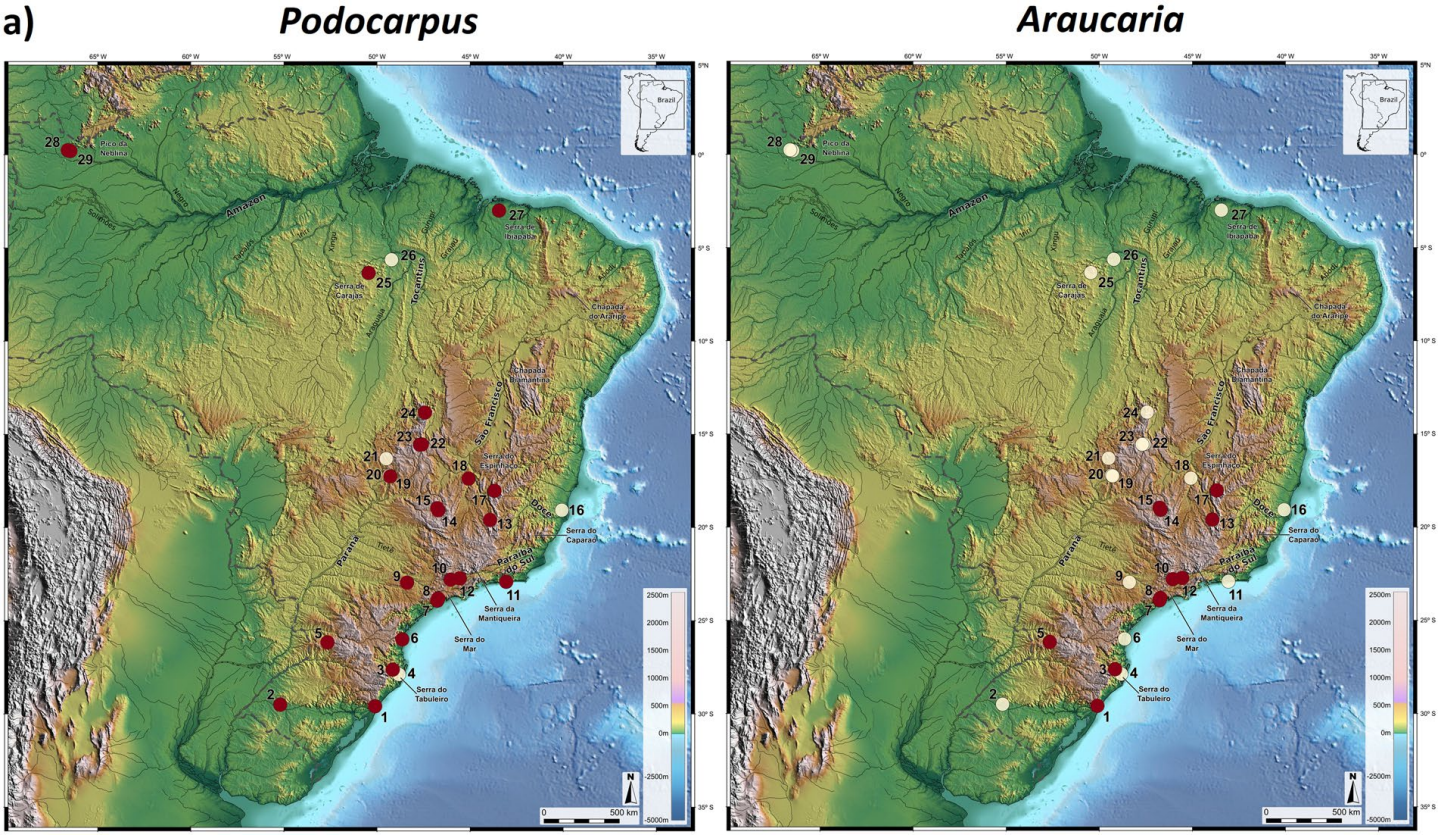
Presence (red circles) and absence (white circles) of *Podocarpus* (**a**) and *Araucaria* (**b**) pollen in HS1 records of Brazil. Areas above 610 m elevation are highlighted in red. Dashed line is the border of Brazil. Base layer: Shaded relief image of ETOPO1 Global DEM (continental area: shaded relief illumination from 060°N, 30° above horizon, 40 times vertical exaggeration; oceanic area: illumination from 060°N, 20° above horizon, 5 times vertical exaggeration).

**Figure 2:**
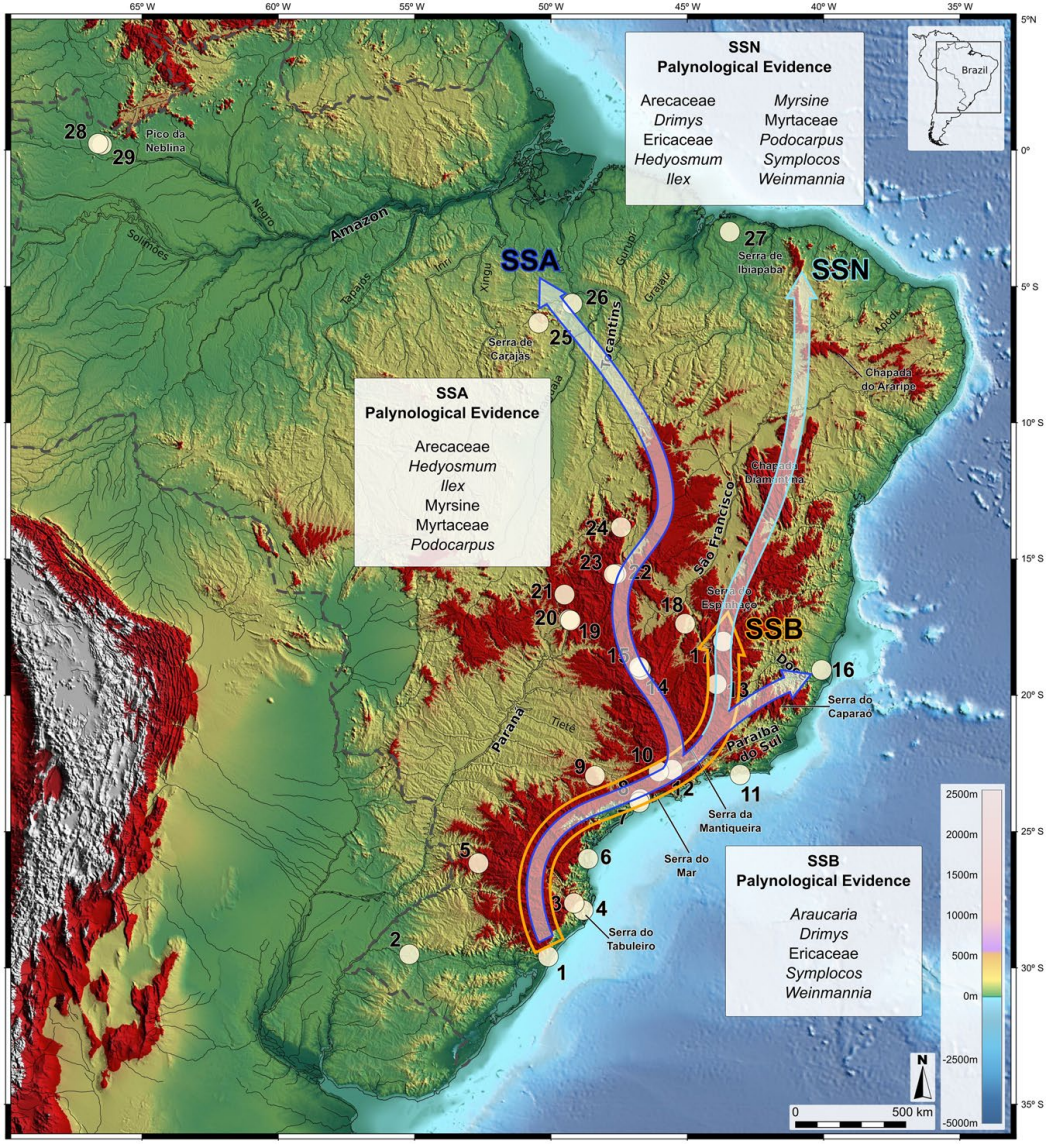
SSA, SSN and SSB migration routes for montane taxa during HS1 and pollen record locations in Brazil (open circles). Route SSA extends from southern/southeastern Brazil to southern Amazonia in the State of Pará. Route SSN extends from Southern-Southeastern to Northeastern Brazil lacks palynological support but is supported by modern distributions of montane taxa and Lagoa do Caçó (sedimentary record 28). SSB route connects coastal southern and southeastern sites up to 18°S synchronous with polar air mass incursions and lowered temperatures as supported by pollen evidence. Areas above 610 m elevation are highlighted in red. Dashed line is the border of Brazil. Base layer: Shaded relief image of ETOPO1 Global DEM (continental area: shaded relief illumination from 060°N, 30° above horizon, 40 times vertical exaggeration; oceanic area: illumination from 060°N, 20° above horizon, 5 times vertical exaggeration).

**Figure 3:**
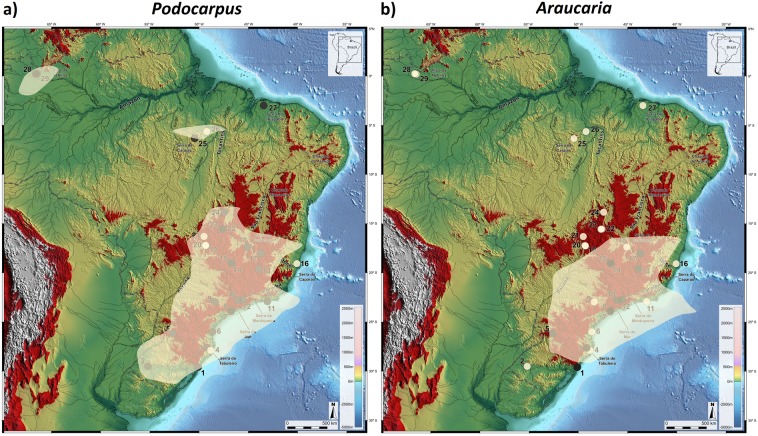
Montane forest potential distribution during HS1 represented by *Podocarpus* (**a**) and *Araucaria* (**b**), where black and white circles indicate presence and absence, respectively, in pollen records. White areas show SDM maps generated in MaxEnt version 3.3.3k for prediction of montane forests, using presence in pollen records during HS1 correlated with climatic layers from CCSM3 Trace21k dataset. Areas above 610 m elevation are highlighted in red. Dashed line is the border of Brazil. Base layer: Shaded relief image of ETOPO1 Global DEM (continental area: shaded relief illumination from 060°N, 30° above horizon, 40 times vertical exaggeration; oceanic area: illumination from 060°N, 20° above horizon, 5 times vertical exaggeration).

To illustrate the distribution of fossil pollen data from the Brazilian Highlands during HS1 we made shaded relief images (Figs. 1–3, 6 and Supplementary Figures) of the ETOPO1 Global Digital Elevation Model^45^ with 01-minute spatial resolution, draped by a custom hypsometric color scale. For the continental area, shaded relief illumination is from 060°N, 30° above horizon, with 40 times vertical exaggeration. In the oceanic area, illumination is from 060°N, 20° above horizon, with 5 times vertical exaggeration. Raster shading and color scale creation^46^ were performed in GRASS-GIS 7.4, map composition in QGIS 3.2 and final artwork in Inkscape 0.92.

Of all 89 pollen records examined in our survey, only 29 encompassed large time sections of the last glacial cycle as shown in Fig. 1 (and in Supplementary Materials), which highlight the two gymospermous taxa of the Brazilian Flora, *Podocarpus* and *Araucaria*, conspicuous elements of southern/southeastern vegetation, during HS1 and indicative of subtropical climates^13,19,25,26^.

### Northward migration during HS1 conditions

The fossil pollen data reveal constant presence of humid forest elements in montane corridors following three major routes during the HS1 event: a northward expansion linking southern-southeastern Brazil to southern Amazonia, via Serra do Mar/Mantiqueira, central Brazil, Serra Geral, Serra de Carajás, hereafter Southern-southeastern Brazil to southern Amazonia (route SSA) and a northward expansion linking southern-southeastern Brazil to northeastern Brazil, via Serra do Mar/Mantiqueira, Serra do Espinhaço, Chapada Diamantina, Serra de Ipiabapa, hereafter Southern-southeastern Brazil to northeastern Brazil (route SSN) and a more restricted distribution connecting southern/southeastern to central Brazil, extending from 30°S to 18°S, hereafter Southern-Southeastern Brazil (route SSB; see Fig. 2, lower right). The SSA and SSN pattern are evident for *Podocarpus*, *Ilex*, *Myrsine*, *Hedyosmum*, Myrtaceae and Arecaceae, describing an arch-like direction. Some of these taxa extended from the southern/southeastern coast at latitudes as low as 30°S, to the central elevated regions of the Brazilian subtropics, thus reaching southeastern Amazonia at 5°S (*Podocarpus, Hedyosmum* and Arecaceae) and 4°S within the modern Cerrado/Amazon rainforest ecotone of northern Maranhão State (Myrtaceae, *Myrsine*) (routes SSA and SSN, respectively; Fig. 2). It is noteworthy that although most species participate in these different pathways, their environmental characteristics are fundamentally different. SSA is associated with the humid and warm Amazonian lowlands, while SSN is associated with xeric environments such as the semi-arid caatinga of the northeast, and SSB is restricted to humid and cool habitats of the southern high-elevation mountains.

We hypothesize that modern plant distributions on isolated mountaintops of northeastern Brazil indicate that various rainforest arboreal taxa^2,47^ possibly reached these regions through a secondary fork-like branch emerging at 22°S and extending until 18°S into the Brazilian highlands, very likely to have been maintained by orographic rains and/or adiabatic cooling. In addition, from 18°S northwards, the SSN route could have reached latitudes within the present-day domain of semi-arid caatinga. Although this additional route during HS1 cannot be confirmed by palynological analyses due to the lack of study sites in that region, macrobotanical evidence in calcareous tufas deposited at ca. 17 kcal yr BP^48^ indicate the presence of Atlantic/Amazonian rainforest elements such as *Aparisthmium* Endl., Annonaceae Juss., Chrysobalanaceae R. Br., *Drymonia coccinea* (Aubl.) Wiehler, *Luehea grandiflora* Mart*., Prunus sellowii* Koehne, *Sloanea* L., *Tetrastylidium* Endl. at 10°S lat. during Heinrich Stadial events in Northeastern Brazil.

Route SSB, utilized by *Araucaria*, *Drimys*, *Symplocos*, *Weinmannia* and Ericaceae, appeared to have had a more restricted distribution in Southeastern Brazil limited within a latitudinal band between 30°S and 18°S. This pathway appears to have two different latitudinal components: a coastal distribution at low elevation from 30°S to 22°S (*Araucaria*, *Drimys*, *Symplocos*, *Weinmannia*, and Ericaceae) and a high montane component from 22°S to 18°S (*Araucaria* and *Drimys*).

All migration pathways suggest that during HS1 a long chain of mountains, starting at the Serra do Mar, followed by the Serra da Mantiqueira and deriving into the Serra do Espinhaço and the Brazilian Central Plateau, functioned as an efficient corridor for the migration of montane elements as proposed by earlier biogeographers^49^, possibly under an enhanced humid phase in the SACZ and ITCZ climate domains^5–7^. Also, the occurrence of cold-adapted trees such as *Araucaria* beyond their present-day northern limit suggests the possibility that intensified polar air incursions into the interior of South America might have dropped the temperatures during the HS1 period as has been inferred for the last glacial period^37,50^. During that time, the tilt of the Earth´s axis, the eccentricity of the orbit and the longitude of the perihelion may have affected the solar radiation at the top of the atmosphere. Therefore, it is possible that under a scenario of less intensified solar radiation during HS1 polar circulation became somewhat stronger while the Hadley circulation was weakened and this mechanism is likely to have generated a larger displacement of polar air towards northern South America^51^.

Under these conditions of enhanced humidity and lowered temperatures, the maintenance of both routes appears to be controlled by different pollen and seed dispersal abilities. Most taxa that migrated further along the SSA route have wind-dispersed pollen and are dioecious, two evolutionary traits directly linked to high dispersal potential^52^, in addition to seed dispersal by birds (Table 1), all of which may have granted them increased ecological amplitude and greater colonization ability. Moreover, these taxa can occur in both late and early successional stages, which can be interpreted as highly adapted to these vegetational changes.

**Table 1:** Reproductive characters of montane arboreal taxa displaying SSA, SSN and SSB migration routes.

Taxon	Routes	Succession	Pollen dispersal	Seed dispersal	Reproduction
*Podocarpus*	SSN-SSA	Late secondary but resistant to disturbance^69^	Anemophilous^70^ (wind)	Zoochory (Birds, mammals)^71,72^	Dioecious
*Ilex*	SSN-SSA	Early and Late^61,73^	Entomophilous^74,75^, Anemophilous^76^	Autochory^77^, Zoochory^77^ (Birds)	Dioecious
*Myrsine*	SSN-SSA	Early and Late^78^ Intermediate^79^	Anemophilous Entomophilous	Zoochory^77,80^(Birds)	Monoecious
*Hedyosmum*	SSN-SSA	Late^81^	Anemophilous^82^	Zoochory (Birds)^82,83^	Dioecious
Myrtaceae	SSN-SSA	Late and early^73^	Entomophilous, Ornithophilous^84^	Zoochory^80,85^ (Bat, bird, small and medium mammals)^84,86^	Monoecious
Arecaceae	SSN-SSA	Early, intermediate or late^87^	Entomophilous, Anemophilous^88^	Zoochory (Bird, small mammals)^84^, Autochory	Monoecious
*Araucaria*	SSB	Early^89^	Anemophilous	Zoochory^89^ (Birds, mammals)	Dioecious
*Drimys*	SSN	Late secondary^90^	Entomophilous^91^	Zoochory (Birds, small mammals)^91^	Dioecious
*Symplocos*	SSN	Late secondary^73^	Entomophilous^92^ Ornithophilous	Zoochory^80^	Monoecious
*Weinmannia*	SSN	Early and secondary^90^	Entomophilous^86,93^	Anemochory^83^	Monoecious
Ericaceae	SSN	Early^94^ and Late^95^	*Gaylussacia –* Entomophilous^93^ *Agarista* Ornitophilous^93^	Zoochory, Autochory, Anemochory^83^	Monoecious

Taxa displaying the SSN route, in general, are characterized by slightly less efficient dispersal of pollen in terms of distance by entomophily as well as zoochory, other than bird-dispersed, seeds. We hypothesize that these these main routes were actually transects of high montane microrefugia alignments during the humid and cold phase of HS1 and probably in earlier glacial phases. Therefore, the small nuclei of montane vegetation acted as sources of immigrants to other microrefugia along theses routes, where all dispersal syndromes were highly efficient, thus permitting geneflow between these populations. Support for this hypothesis comes from a modernal relictual *Podocarpus* microrefuge in semi-arid vegetation at Morro do Chapeu, Bahia at 11°S^53,54^. There, rocky otucrops reduce mean annual temperature by 5 °C and augment humidity by 12%. Under this scenario, final coalescence of these microrefugia may have formed rather contiguous humid forested corridors. Testing these hypotheses requires more pollen records especially at lower latitudes of southern Amazonia and northeastern Brazil. This paleoclimatic scenario is supported by a marine pollen record off the coast of northeastern Brazil^55^, containing continental sediments generated in the region of modern semi-arid vegetation. This record shows an HS1 pollen zone, characteristic of humid climate, with significant percentages of SSA route taxa such as *Hedyosmum*, *Ilex*, and Myrtaceae, together with *Symplocos* and *Cyathea* Sm. It is clear that entomophilous pollination and animal-dispersed seeds did not hinder the very long dispersal ability of *Drimys* possibly due to the beneficial effects of nearby microrefugia and therefore shorter dispersal distances needed for population expansion. The data suggest that the SSN route could have extended well into northeastern Brazil, where *Drimys*, *Hedyosmum*, *Symplocos*, Ericaceae, *Podocarpus*, *Myrsine*, *Ilex*, *Weinmannia* and others are all found at the modern altitude of 1500 m in the Chapada Diamantina range, in northeastern Brazil.

The main feature of route SSB is the northward expansion of *Araucaria*, a major element in ombrophilous rainforest of high elevations of southern/southeastern Brazil in consortium with *Podocarpus*. It can be considered a good proxy for relatively low temperature and high humidity^12,13,27,56,57^. Our hypothesis suggests that its migration pattern reaching 18°S was likely to be controlled by low temperature regimes and low precipitation variability throughout the year during HS1. Although low modern temperature regimes at the Serra do Espinhaço (site 17) are conducive to sustaining gymnosperm forests as they did during HS1, the site no longer has adequate humidity due to irregular distribution of precipitation during the long dry season.

Hereafter represented by *Podocarpus*, demonstrate wider latitudinal expansion abilities, ranging from 30°S to 0°S, than those displayed by SSB, hereafter represented by *Araucaria* (Supplementary Figs. 1–11), where taxa were restricted to latitudes between 30°S and 18°S.

### Climatic changes and montane vegetation expansion during HS1

Pollen histories of arboreal taxa common in humid and cold forests of glacial age in Brazil^12,13,23,37,40^ support the hypothesis of intensification of transient climatic systems under glacial regime during HS1. We suggest that continental surface temperatures lowered by the effects of polar air incursions in South America had a significant forcing effect on modern plant biogeographical patterns by fostering long-distance migration of currently montane elements. One of the best-known lines of indirect evidence for this hypothesis is given by the vegetation of the Pico das Almas (13°34′S), in the Chapada Diamantina mountain range, northeastern Brazil. This vegetation is floristically more related to that of the Andean paramo and subparamo forests^2^ with genera like *Podocarpus*, *Drimys*, *Symplocos*, *Weinmannia*, and *Hedyosmum* (SSA pattern), and to the flora of the tepuis of Venezuela. The second line of indirect evidence is the pollen record of the Icatu site at 10°S, currently under semi-arid vegetation in northeastern Brazil, which shows during the Pleistocene/Holocene transition^58^ at c. 11 kcal yr BP abundant cold and humid-adapted taxa such as *Podocarpus*, *Ilex*, Myrtaceae and *Hedyosmum* coexisting with *Humiria* Aubl., a taxon of shrubs to large trees of the Guyanas and Guyana-influenced Amazon and currently present at Serra do Espinhaço range above 1000 m altitude^59^.

Although alternative and viable hypotheses for such biogeographical patterns may suggest that connections could have been established during the cold phases of the Oligocene^1,2^, following the tropical decline of the Eocene, Late Pleistocene pollen data points to a powerful reorganization of ecosystems in South America during the terminal phases of the last glacial cycle. To test this Late Quaternary expansion of cold and humid forests, *i.e*. augmentation of the potential distribution of the fundamental niche of our selected montane taxa into northern Brazil, we generated a Species Distribution Model (SDM) for *Podocarpus* and *Araucaria* for the HS1 phase in the Brazilian Highlands, by correlating observations of taxa occurrences in palynological records, representatives of the SSA, SSN and SSB routes, with monthly mean of precipitation and surface temperature from climatic layers from the CCSM3 Trace21k dataset^44^ (Fig. 3).

**Figure 4:**
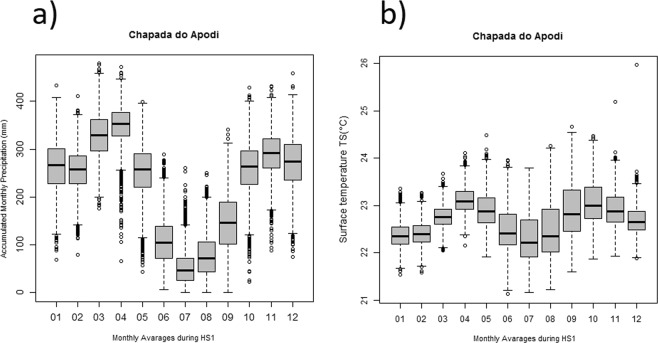
Boxplot of monthly averages of convective precipitation rate (PRECC) in mm/month (**a**) and mean monthly surface temperature TS °C (**b**) at Chapada do Apodi, next to Caçó Lake, derived from Simulation of the Transient Climate of the Last 21000 Years (TraCE-21k) during HS1. R-scripts were generated to produce boxplots.

The resulting maps highlight areas of habitat suitability over large sections of the SSA and SSB routes especially those between 30°S and 14°S, a region where the South American Monsoonal System was intensified during HS1^6,7,60^. The disjunct high suitability for *Podocarpus* for the Carajás lakes region (site 25) and very low suitability for Lagoa do Caçó^41^ (site 27) might be due to the absence of continental pollen records in these equatorial and subequatorial regions in Brazil during HS1. On the other hand, a marine pollen record off the coast of Ceará^61^, showing expansion of cold-adapted montane arboreal elements during HS1, provide strong evidence for high humidity levels brought about by the southward displacement of the ITCZ^6^. This scenario is confirmed by CCSM3 Trace21k dataset^44^ simulation data analyses for current semi-arid Apodi region (<250 m elevation, mean annual temperature 28.5 °C), nearby Caçó lake, indicating average annual accumulation of 2650 mm associated with high precipitation levels of monthly mean Convective Precipitation Rate (PRECC) and mean annual surface temperatures (TS) of around 22.6 °C, of c. during HS1 and (Fig. 4). Therefore, we estimate a temperature depression of c. 5 °C, which is supported by similar cooling between ca. 14–17 Krs BP, revealed by noble gases paleotemperature record in groundwater in northeastern Brazil^62^. In addition to conducive temperature, precipitation values are also well above the ecological requirements for sustaining a cold and humid forest vegetation with *Podocarpus* at Caco Lake, currently under mean annual precipitation of 1400–1500 mm and mean annual temperature of 25 °C^38^.

**Figure 5:**
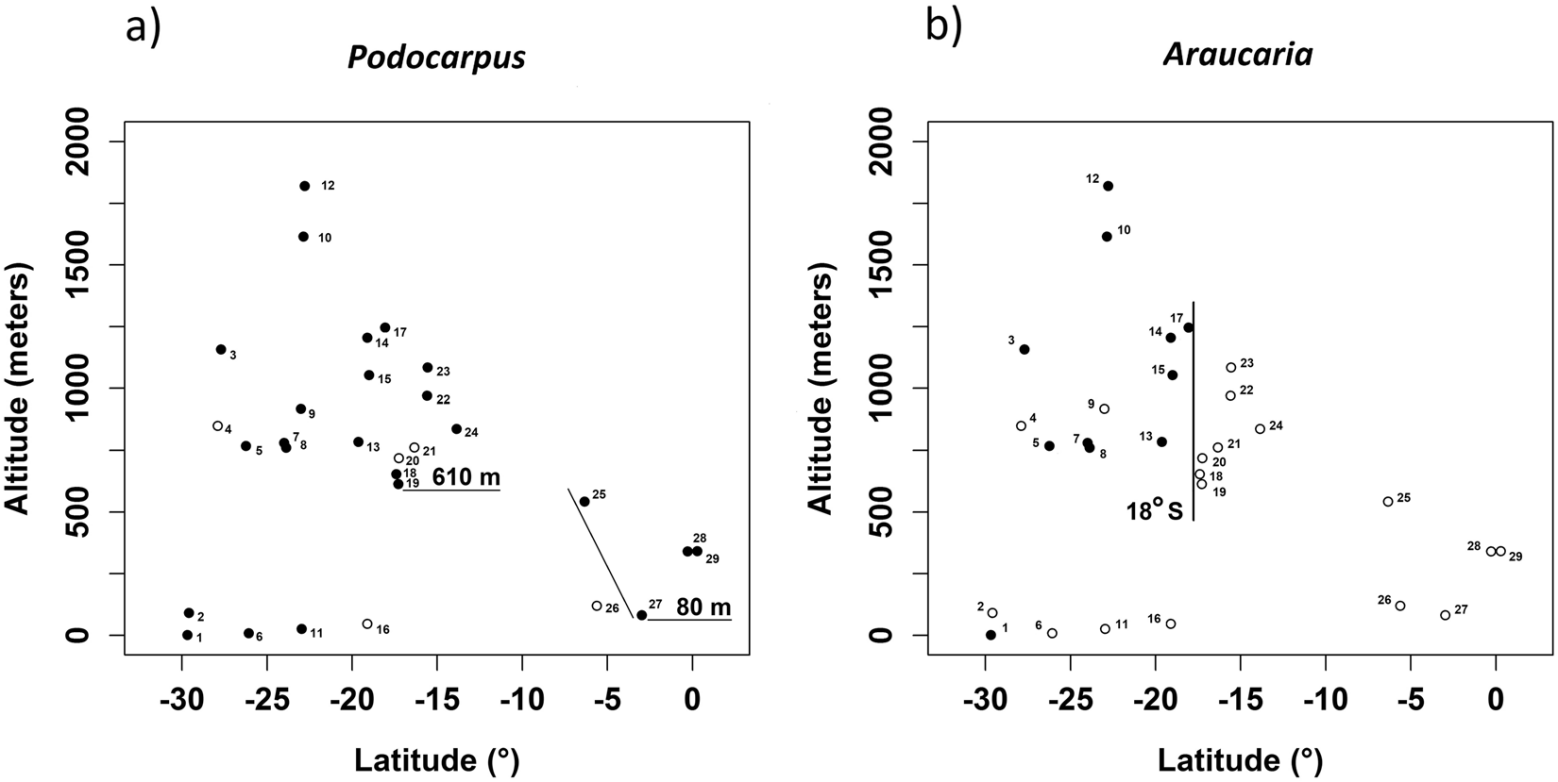
Scatter plot of altitude (m) vs. latitude (°S) for *Podocarpus* (**a**) and *Araucaria* (**b**) as indicated by presence (filled circles) and absence (clear circles) in pollen records during HS1. R-scripts were generated to produce the scatter plot.

The frequency of *Podocarpus* in pollen records analyzed depict a strong latitudinal control. In the southernmost range of its distribution (30°S-22°S) it could be found in elevations ranging from sea level to 1157 m, followed by an intermediate range from north of 22°S to 10°S where it was restricted to elevations higher than 610 m (Fig. 5a). In its northernmost limit, lying between 6°S and 3°S, *Podocarpus* eventually reached the Amazonian lowlands, thus descending from 540 m to 80 m elevation (Fig. 5a, lower right), next to Caçó Lake. *Araucaria*, on the other hand, a good representative of the SSB route, shows a dissimilar distribution pattern in the HS1 pollen records ranging from 30°S at sea level to 750 m at 26°S with its northern limit during the glacial times at 18°S in 1246 m elevation, with maximum elevation reached at 1820 m at 22°S. The separating line between sites north and south of 18°S (Fig. 5b) suggests the northernmost limit for incursion of polar air masses during HS1, a fact that possibly imprinted a biogeographical boundary still observed in the modern southeastern Atlantic rainforest with a higher plant species turnover^63^ north of 18°S. Molecular genetic data from three common species of tree frogs widely distributed along the Atlantic rainforest^64^ support forest stability in the central core area of this vegetation type in the late Pleistocene. Combining these with our results, it becomes clear that stability of forest physiognomic persisted during the Late Pleistocene concurrent with migration of cold-adapted plant species northwards propelled by H1 events, which in turn might have contributed to the establishment of biogeographical compartmentalization of the montane coastal vegetation specially north of 18°S.

### Modern parameters and the climate of HS1 for the brazilian highlands

**Figure 6:**
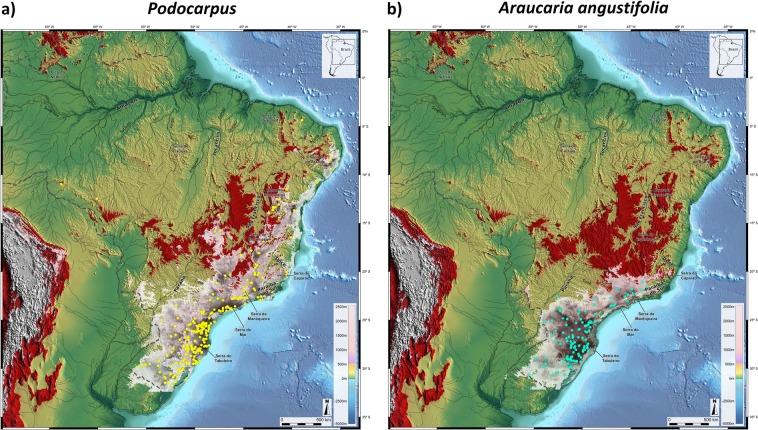
Modern Potential Distribution maps for *Podocarpus* (**a**) and *Araucaria angustifolia* (**b**) where occurrences of taxa are shown by yellow and blue dots, respectively. White areas show SDM maps generated by MaxEnt version 3.3.3k for prediction of montane forest, using presence from SpeciesLink and SiBBr/GBIF database and 19 bioclimatic data layers from WorldClim dataset version 2.0. Areas above 610 m elevation are highlighted in red. Dashed line is the border of Brazil. Base layer: Shaded relief image of ETOPO1 Global DEM (continental area: shaded relief illumination from 060°N, 30° above horizon, 40 times vertical exaggeration; oceanic area: illumination from 060°N, 20° above horizon, 5 times vertical exaggeration).

*Podocarpus* L’ Her. ex Pers. and *Araucaria* (Bertol.) Kuntze stand out for beeing more ecologically informative in terms of precipitation and temperature requirements in southern and southeastern Brazil. Species Distribution Model (SDM) maps for *A. angustifolia*, *P. lambertii and P. sellowii*, shown in Fig. 6, confirm a geographical restriction of *Araucaria* in southern/southeastern Brazil as opposed to a large distribution of *Podocarpus*, ranging rom 30°S to ca 5°S. Data cleaning methods was used to check the quality of data of modern distribution^42^, i.e. validation of the taxonomic identification in relation to the available literature with its corresponded latitude and longitude coordinates was performed. Georeferencing errors were evaluated and discarded from the database and a filter was applied to select only occurrences within the Brazilian territory.

At the northernmost edge of this distribution range, *Podocarpus* is found in relictual populations in orographically controlled humid “islands” within semi-arid climates^53,54^, isolated in the Pleistocene, as indicated by genetic data^65^. Anotherline of evidence for expanded montane forest in this region is the discovery^9,48^ of fossilized leaves of rainforest taxa found in the late glacial calcareous tufas in a modern Caatinga region adds more support for this climatic mechanism during Heinrich Stadial events throughout the last glacial period. However, the mode that humid adapted plants migrated to present-day semi-arid regions in South America is still unknown.

The current distribution of *Araucaria angustifolia* reaches its northern limit at 21°S in any elevation, at altitudes of about 400 to 1750 m above sea level (asl) under less than three months dry season, whereas from 24° to 31°S this taxon occurs as low as at sea level up to ca. 1200 m, under year-round high humidity levels.

In order to investigate the factors controlling its modern distribution as well as *Podocarpus lambertii* occurrences, we used a 15-year long data set of rainfall data CPC Morphing technique (CMORPH)^66^, corrected by the Brazilian meteorological station network data^67,68^, between 2000 and 2015. Afterwards, R-scripts were written to generate descriptive statistics for the occurrence of each taxon. Box plots and histograms were generated based on values of 1° × 1° latitude grid of total daily accumulated precipitation data. These analyses yielded mean values of precipitation for 6 months periods, for the seasons, months and annual precipitation for that historic series.

Finally, a Principal Component Analysis (PCA) was carried out for both *Podocarpus* and *Araucaria* in order to discriminate the roles of precipitation and temperature in relation to the following variables: cumulative annual precipitation in mm, total precipitation in Winter, Spring, Summer and Autunm, wet and dry periods. Modern occurrence of *Araucaria angustifolia* and *Podocarpus lambertii* in Brazil are controlled mainly by mean total annual precipitation of 1680 ± 180 mm and 1520 + 220 mm based on a 15-year climatic series (2000–2015), whereas optimum mean annual temperature for both taxa are ca. 17.5 °C as indicated by Species Distribution Model and WorldClim dataset.

**Figure 7:**
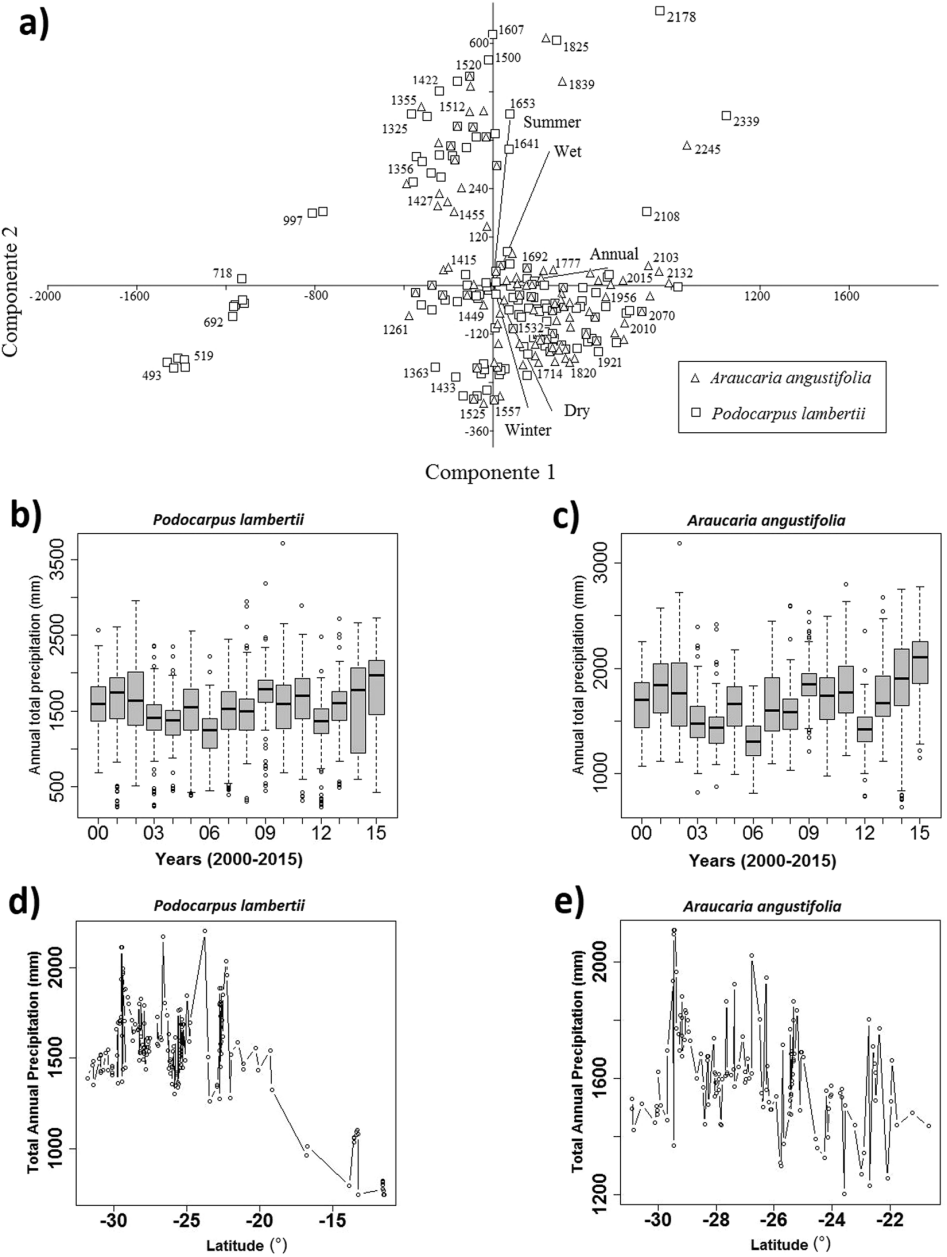
PCA biplot for the modern distribution of *Podocarpus lambertii* and *Araucaria angustifolia* in relation to mean values of total annual precipitation, performed by PAST 3.21 (**a**). Boxplots of each annual total precipitation value representing number of years (2000–2015) for *Podocarpus lambertii* (1520 ± 220 mm) (**b**) and *Araucaria angustifolia* (1680 ± 180 mm) (**c**). Scatter plot of Total Annual Precipitation (mm) vs. Latitude (°) of modern distribution for *Podocarpus lambertii* and *Araucaria angustifolia*, respectively (**d,e**). Precipitation data were based on hourly rainfall estimates with CMORPH between 2000 and 2015, corrected by the Brazilian meteorological station network. R-scripts were generated to produce boxplots and the scatter plot.

PCA biplots shown on Fig. 7 indicate that annual and summer variables are the main loading values for the PC1 (84.11%) and PC2 (15.15%) components, respectively. The simultaneous occurrence of *Araucaria angustifolia* and *Podocarpus lambertii* is discernibed as a main central cluster (Fig. 7a) for 1750 mm annual precipitation. Outside this range, only *Podocarpus* can survive as it tolerates higher precipitation variability (Fig. 7b). The latitudinal control for *Araucaria* is significantly stronger when compared to *Podocarpus* that can thrive in microrefugia in latitudes outside the *Araucaria*-*Podocarpus* associations typical of south/southeastern Brazil (Fig. 7d,e). The PCA diagram of the combined distribution, with overlapping clusters, of both taxa shows that the modern occurrences of *Araucaria angustifolia* and *Podocarpus lambertii* in Brazil are controlled mainly by mean total annual precipitation of 1680 ± 180 mm and 1520 ± 220 mm respectively based on a 15-year climatic series (2000–2015). These values range well within the classical precipitation values used in the literature for both taxa for the Brazilian highlands.

**Figure 8:**
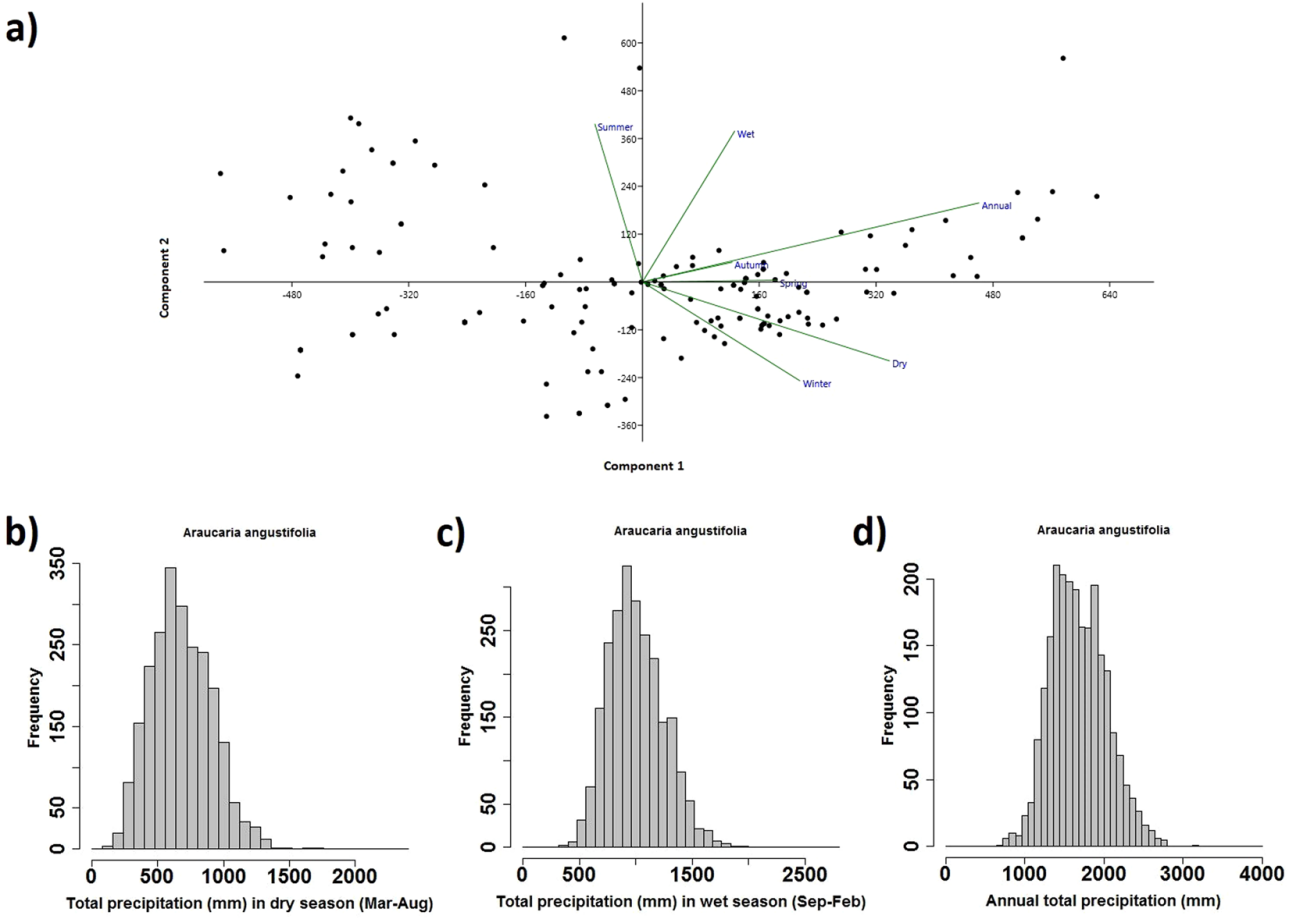
PCA biplot (**a**) for modern distribution of *Araucaria angustifolia* in relation to mean values of total annual precipitation, performed by PAST 3.21. Annual (**b**), wet (**c**) and dry (**d**) season histograms for total precipitation values representing number of years (2000–2015), in relation to mean values of total monthly precipitation. Precipitation data were based on hourly rainfall estimates with CMORPH between 2000 and 2015, corrected by the Brazilian meteorological station network. R-scripts were generated to produce boxplots and the scatter plot.

PCA biplots for *Araucaria* (Fig. 8a) reveals annual and summer variables are the main loading values for the PC1 (71.47%) and PC2 (27.07%) component. PCA loadings indicate that the distribution of *Araucaria angustifolia* is primarily influenced by anual acumulated precipitation and well distributed precipitation long the year, both in dry and wet seasons, respectively. Figures 8b,c indicates low precipitation variability as a significant parameter for explaining *Araucaria* distribution (Fig. 8d) under modern conditions. Therefore, it’s clear that this taxon does not tolerate dry conditions even during the dry months, showing an optimum value of precipitation around 500 mm for the period of March to August.

We suggest that individualistic species reshuffling in the three migration routes during HS1 was constrained by their physiological responses within these temperature and precipitation ranges. Morevorer, the climatic confinment in terms of humidity stability possibly explains why *Araucaria* did not migrate further than lat 18°S in route SSB during HS1.

## Conclusions

Our analyses provide strong evidence for the establishment of migration corridors of montane forest taxa, facialtitated by the presence of microrefugia, connecting different ecosystems in a continental scale, during humid and cold conditions related to Heinrich Stadial 1, from 18.1 to 14.7 kcal yr BP. Analysis of published Brazilian fossil pollen records corroborate with a climatological scenario of high humidity sustained by the South American Monsoonal System under lowered temperatures resulting in more regular polar air mass incursions, reaching 18°S latitude, thus forming frequent cold fronts advancing northwards, into Amazonia and northeastern Brazil. Consequently, the two main migration routes, here coined SSA and SSN, respectively, were established. On those montane pathways, different plant taxa were able to disperse taking full advantage of certain reproductive traits, especially anemophilous pollen and bird dispersed seeds. It’s possible, that effective migration along these routes were facilitated by the presence of previous montane forests microrefuges, which might have expanded downslope towards the lowlands during HS1, thus forming coalesced vegetated corridors. The proximity bwtween these established microrefugia permitted a series of local population expansions along the three montane routes during these cold and humid events.

It becomes clear that mean annual temperature depression played a major role during the wet and cold HS1 phase in Brazil of at least 5 °C, as indicated by temperature estimation based on noble gas paleotemperature records not for Last Glacial Maximum but also for HS1^62^. In addition, the presence of modern humid microrefuge containing montate taxa such as *Poducarpus*^53^ within hyper-xerophylous caatinga is indicating a previous wetter climate conditions that took place during the establishment of SSN route.

The Brazilian highlands latitudinal range of 30°S to 18°S, conducive to expansion of montane vegetation with *Araucaria* during HS1, has probable ecological limits controlled by temperature. However, in its northern boundary of the range, intense monsoon rainfall caused shorter seasonality in comparison to its current limit at 21°S while mean annual temperatures are today approximately similar. Currently, at 18°S latitude, long dry seasons of 5 to 6 months inhibit the growth of *Araucaria angustifolia*. In comparison, its fossil pollen occurrence during HS1 implies in a well-distributed precipitation throughout the year. By integrating the pollen data with speleothem isotope records^3,5,7^, we can assume that South American Monsoon regime during HS1 was longer with higher annual precipitation rates.

Unlike *Araucaria* and other taxa that followed the SSB migration route, *Podocarpus* and others were able to migrate further north and beyond the highland domain, thus reaching distant lowlands regions due to their ability to disperse pollen and seeds more efficiently, and to germinate and grow in understory and dark forests. Their enlarged biogeographical range during HS1 primarily reflects a close approximation of their fundamental niches in opposition to their realized niche after the onset of Holocene warm climates. This significant habitat reduction is likely to have established modern disjunct distribution patterns between southern/southeastern and northeastern Brazil.

We, therefore, conclude that, although we do not devalue a possible scenario of early migration corridors during the Eocene/Miocene^1,2^ of Brazil, a significant imprint of the HS1 in delineating modern disjunct distributions there is unquestionable.

One of the major implications of this study deals with possible impact of rising temperature predicted for future climatic scenarios on a substantial loss of tropical montane biodiversity. Our investigation suggests that the change in rainfall distribution may potentialy enhance this process.

## Supplementary information

Supplementary Informations
