# Supplementary material for: Brazilian montane rainforest expansion induced by Heinrich Stadial 1 event
Source: Sci Rep. 2019 Nov 29;9:17912. doi: 10.1038/s41598-019-53036-1 (PMC6884455; doi:10.1038/s41598-019-53036-1)
Supplement: Supplementary file 1 — Supplementary Informations [file 41598_2019_53036_MOESM1_ESM.pdf]

Supplementary Information

Brazilian montane rainforest expansion induced by Heinrich Stadial 1 event

Jorge L. D. Pinaya[1*], Francisco W. Cruz[2], Gregório C. T. Ceccantini[4], Pedro L. P. Corrêa[1], Nigel Pitman[3], Felipe Vemado[5], Maria del Carmen S. Lopez[5], Augusto J. Pereira Filho[5], Carlos H. Grohmann[6], Cristiano M. Chiessi[7], Nicolás M. Strikis[8], Ingrid Horák-Terra[9], Walter H. L. Pinaya[10], Vanda B. de Medeiros[2], Rudney de A. Santos[2], Thomas K. Akabane[2], Maicon A. Silva[4], Rachid Cheddadi[11], Mark Bush[12], Alexandra-Jane Henrot[13], Louis François[13], Alain Hambuckers[14], Frédéric Boyer[15], Matthieu Carré[16], Eric Coissac[15], Francesco Ficetola[15], Kangyou Huang[17], Anne-Marie Lézine[16], Majda Nourelbait[11], Ali Rhoujjati[18], Pierre Taberlet[15], Fausto Sarmiento[19], Daniel Abel-Schaad[20], Francisca Alba-Sánchez[20], Zhuo Zheng[17], Paulo E. De Oliveira[2, 3].

[1]Politechnical School, University of São Paulo, São Paulo, SP, Brazil, [2]Institute of Geosciences, University of São Paulo, São Paulo, SP, Brazil, [3]Science Action, The Field Museum of Natural History, Chicago, Illinois, USA, [4]Institute of Biosciences, University of São Paulo. São Paulo, SP, Brazil, [5]Institute of Astronomy and Geophysics and Atmospheric Sciences, University of São Paulo, SP, Brazil, [6]Institute of Energy and Environment, University of São Paulo, SP, Brazil, [7]Laboratório de Paleoceanografia e Paleoclimatologia da Escola de Artes, Ciências e Humanidades, University of São Paulo, SP, Brazil, [8]Federal Fluminense University, Niteroi, RJ, Brazil, [9]Instituto de Ciências Agrárias, Universidade Federal dos Vales do Jequitinhonha e Mucuri, Unaí, MG, Brazil, [10]Center of Mathematics, Computation, and Cognition. Universidade Federal do ABC, Santo André, SP, Brazil, [11]ISEM, Université de Montpellier, Centre National de la Recherche Scientifique, IRD, EPHE, Montpellier, France, [12]Department of Biological Sciences, Florida Institute of Technology, Melbourne, FL, United States, [13]Unité de Modélisation du Climat et des Cycles Biogéochimiques, UR-SPHERES, University of Liège, Liège, Belgium, [14]Behavioural Biology Unit, UR-SPHERES, University of Liège, Liège, Belgium, [15]Laboratoire d'Ecologie Alpine, Centre National de la Recherche Scientifique, Université Grenoble Alpes, Grenoble, France, [16]LOCEAN Laboratory, Sorbonne Universités (UPMC), CNRS, IRD, MNHN, Paris, France, [17]School of Earth Science and Geological Engineering, Sun Yat-sen University, Guangzhou, China, [18]Laboratoire Géoressources, Unité de Recherche Associée CNRST (URAC 42), Faculté des Sciences et Techniques, Université Cadi Ayyad, Marrakech, Morocco, [19]Neotropical Montology Collaboratory, Department of Geography, University of Georgia, Athens, GA, United States, [20]Department of Botany, Faculty of Sciences, Universidad de Granada, Granada, Spain.

List of montane taxa belonging to Myrtaceae Juss., Ericaceae Juss. and Arecaceae Bercht. & J. Presl (*sin*. Palmae Juss., palms) found in humid and cold habitats in Brazil:

Family Myrtaceae Juss. is represented in the Brazilian Highlands by a total of 17 genera and 53 species, as follows: *Blepharocalyx salicifolius* (Kunth) O. Berg, *Calycorectes acutatus* (Miq.) Toledo, *Calyptranthes clusiifolia* (Miq.) O. Berg, *Calyptranthes concinna* DC., *Calyptranthes grandifolia* O. Berg, *Campomanesia eugenioides* (Cambess.) D. Legrand, *Campomanesia guazumifolia* (Cambess.) O. Berg, *Campomanesia hirsuta* Gardner, *Campomanesia laurifolia* Gardner, *Campomanesia neriiflora* (O. Berg) Nied., *Campomanesia xanthocarpa* (Mart.) O.Berg, *Eugenia brasiliensis* Lam., *Eugenia cerasiflora* Miq., *Eugenia glazioviana* Kiaersk, *Eugenia involucrata* DC., *Eugenia macrosperma* DC., *Eugenia multicostata* D. Legrand, *Eugenia pluriflora* DC., *Eugenia rostrifolia* D. Legrand, *Eugenia schuechiana* O. Berg, *Eugenia sonderiana* O. Berg, *Eugenia sprengelii* DC., *Eugenia xiriricana* Mattos, *Feijoa sellowiana* Berg, *Gomidesia affinis* (Cambess.) D. Legrand, *Gomidesia lindeniana* O. Berg, *Gomidesia sellowiana* O. Berg, *Mosiera prismatica* (D. Legrand) Landrum, *Myrceugenia miersiana* (Gardner) D. Legrand & Kausel, *Myrceugenia regnelliana* (O. Berg) Legrand & Kaus, *Myrcia crassifolia* Kiaersk., *Myrcia glabra* (O. Berg) D. Legrand, *Myrcia rostrata* DC., *Myrcia tomentosa* (Aubl.) DC., *Myrcianthes pungens* (O. Berg) D. Legrand, *Myrciaria cauliflora* (Mart.) O. Berg, *Myrciaria coronata* Mattos, *Myrciaria cuspidata* O. Berg, *Myrciaria delicatula* (DC.) O. Berg, *Myrciaria floribunda* (H. West ex Willd.) O. Berg, *Myrciaria disticha* O. Berg, *Myrciaria glazioviana* (Kiaersk.) G.M. Barroso ex Sobral, *Myrciaria grandifolia* Mattos, *Myrciaria phitrantha* (Kiaersk.) Mattos, *Myrciaria strigipes* O. Berg, *Myrciaria tenella* (DC.) O. Berg, *Myrciaria trunciflora* O. Berg, *Neomitranthes gemballae* (D. Legrand) D. Legrand, *Pimenta pseudocaryophyllus* (Gomes) Landrum, *Plinia rivularis* (Cambess.) Rotman, *Psidium oblongatum* O. Berg, *Siphoneugena densiflora* O. Berg, *Siphoneugena widgreniana* O. Berg.

Ericaceae Juss., an almost exclusive montane family of shrubs and trees, is represented in the Brazilian Highlands by 12 genera, 99 species and 27 varieties. Very widespread arboreal taxa of montane forests are *Agarista coriifolia* (Thunb.) Hook. ex Nied., *Agarista oelifoilia* (Cham.) G. Don var. *glabra* (Meisn.) Sleumer, *Gaylussacia brasiliensis* (Spreng.) Meisn. var. nervosa Meisn., *Gaylussacia densa* Cham., *Gaylussacia harleyi* Kin.-Gouv., *Gaylussacia incana* Cham. & Schltdl. and *Gaylussacia reticulata* Mart. Ex Meisn. var. *salviifolia* Sleumer.

Family Arecaceae Bercht. & J. Presl (sin. Palmae Juss., palms), is represented in Brazilian montane forests with subtropical humid climate, with occasional sub-zero temperatures, by 5 genera and 19 species, as follows: *Acrocomia aculeata* (Jacq.) Lodd., *Arecastrum romanzoffianum* (Cham.) Becc., *Butia archeri* (Glassman) Glassman, *Butia eriospatha* (Mart. ex Drude) Becc., *Butia exilata* Deble & Marchiori, *Butia lallemantii* Deble & Marchiori, *Butia microspadix* Burret, *Butia yatay* (Mart.) Becc., *Geonoma blanchetiana* H. Wendl., *Geonoma brevispatha* Barb. Rodr., *Geonoma gamiova* Barb Rodr., *Geonoma maxima* (Poit.) Kunth, *Geonoma schottiana* Mart., *Syagrus flexuosa* (Mart.) Becc., *Syagrus oleracea* (Mart.) Becc., *Syagrus pleioclada* Burret, *Syagrus romanzoffiana* (Cham.) Glassman, *Syagrus sancona* (Kunth) H. Karst., *Syagrus werdermannii* Burret.

Podocarpus

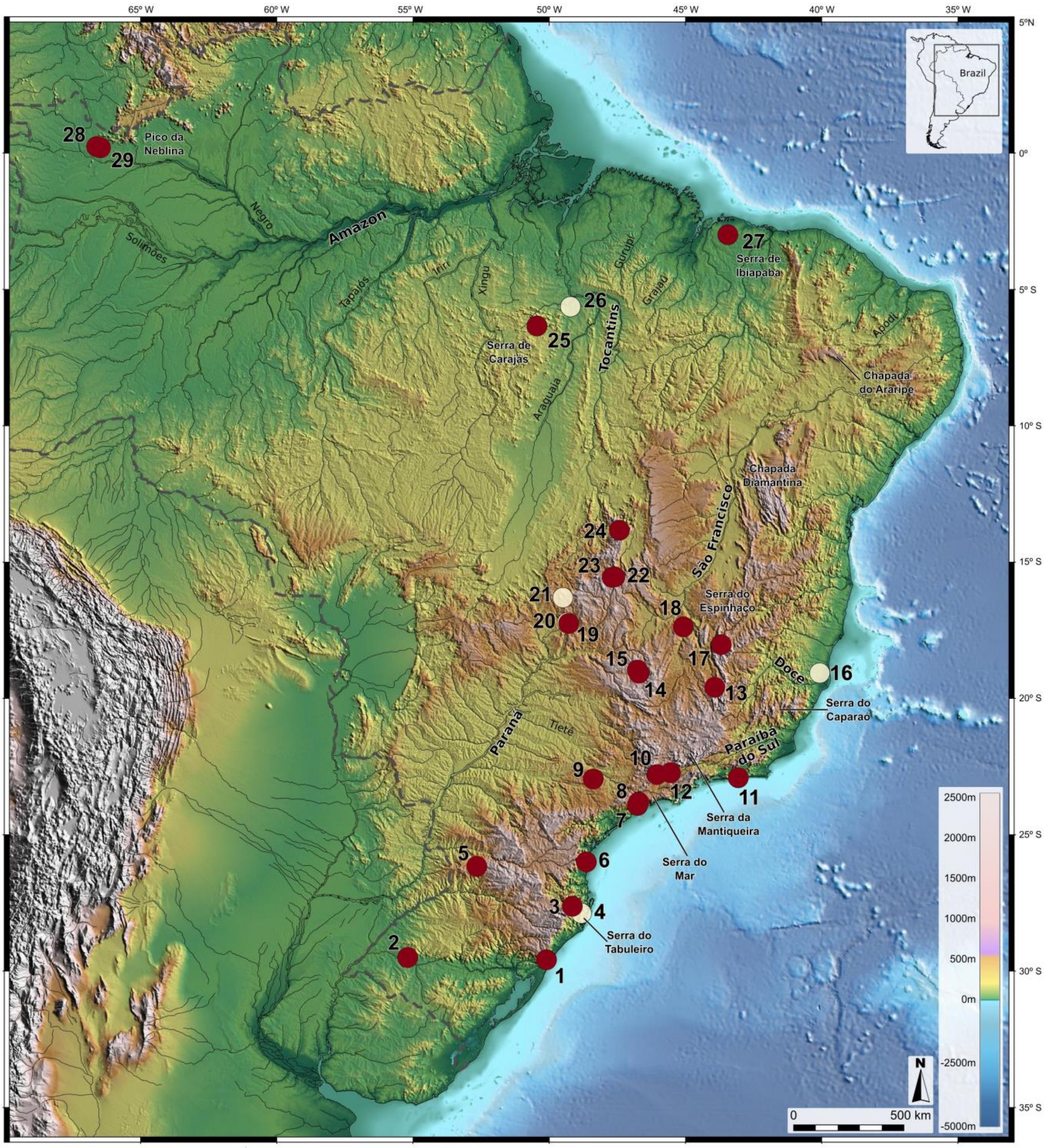

Supplementary Figure 1: Presence (red circles) and absence (clear circles) of *Podocarpus* pollen in HS1 records of Brazil. Base layer: Shaded relief image of ETOPO1 Global DEM (continental area: shaded relief illumination from 060˚N, 30˚ above horizon, 40 times vertical exaggeration; oceanic area: illumination from 060˚N, 20˚ above horizon, 5 times vertical exaggeration).

Ilex

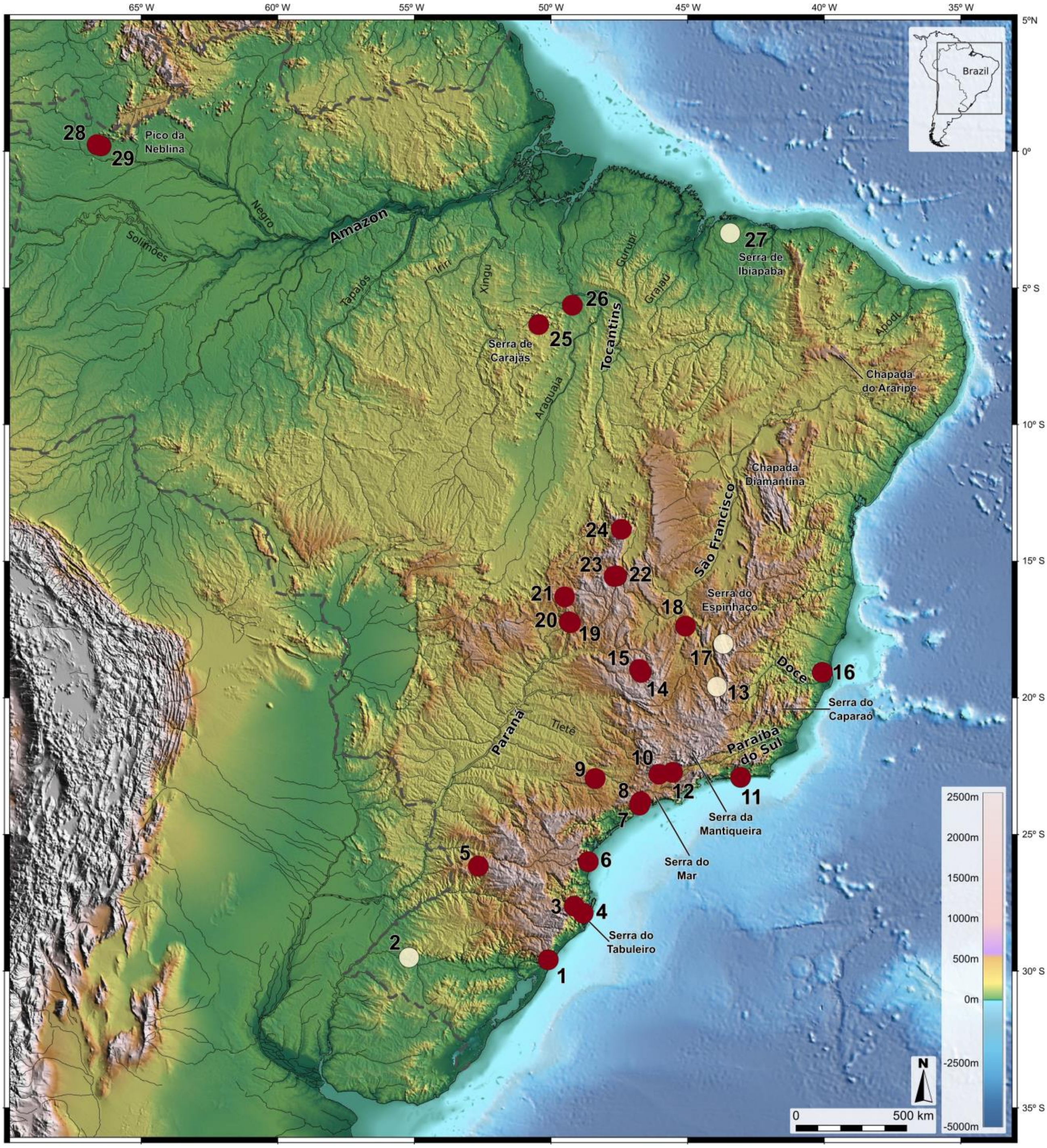

Supplementary Figure 2: Presence (red circles) and absence (clear circles) of *Ilex* pollen in HS1 records of Brazil. Base layer: Shaded relief image of ETOPO1 Global DEM (continental area: shaded relief illumination from 060˚N, 30˚ above horizon, 40 times vertical exaggeration; oceanic area: illumination from 060˚N, 20˚ above horizon, 5 times vertical exaggeration).

Myrsine

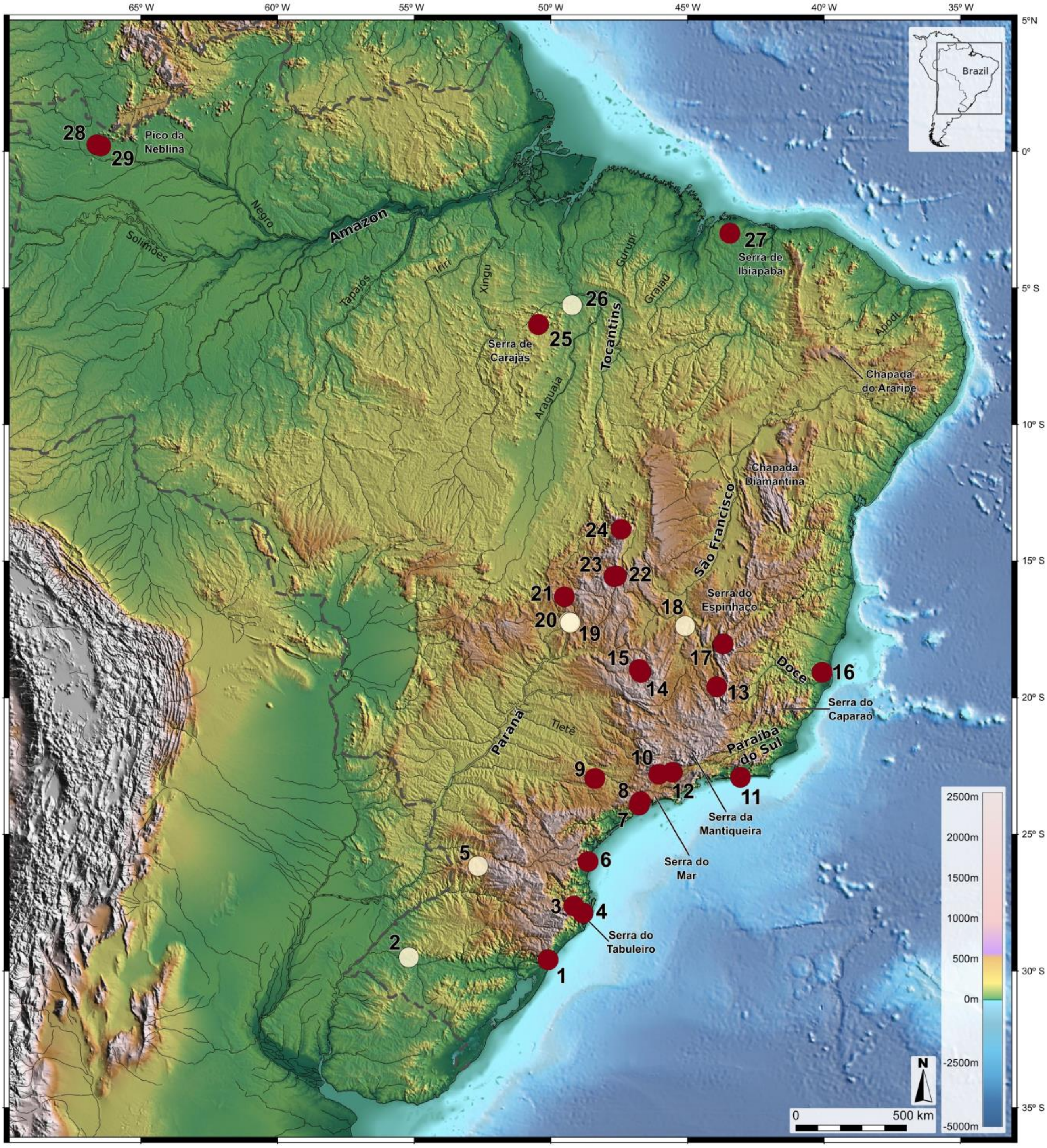

Supplementary Figure 3: Presence (red circles) and absence (clear circles) of *Myrsine* pollen in HS1 records of Brazil. Base layer: Shaded relief image of ETOPO1 Global DEM (continental area: shaded relief illumination from 060˚N, 30˚ above horizon, 40 times vertical exaggeration; oceanic area: illumination from 060˚N, 20˚ above horizon, 5 times vertical exaggeration).

Hedyosmum

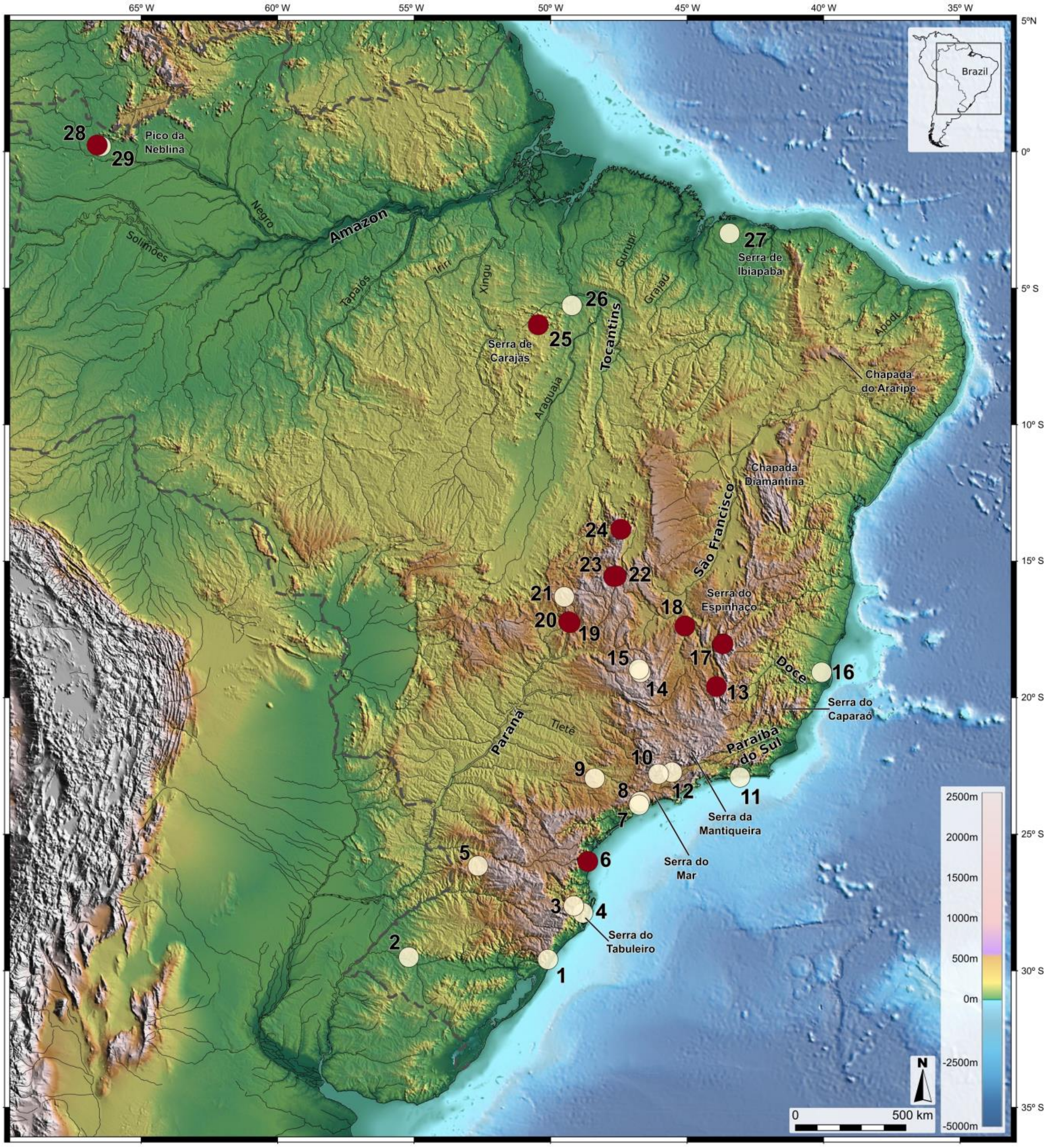

Supplementary Figure 4: Presence (red circles) and absence (clear circles) of *Hedyosmum* pollen in HS1 records of Brazil. Base layer: Shaded relief image of ETOPO1 Global DEM (continental area: shaded relief illumination from 060˚N, 30˚ above horizon, 40 times vertical exaggeration; oceanic area: illumination from 060˚N, 20˚ above horizon, 5 times vertical exaggeration).

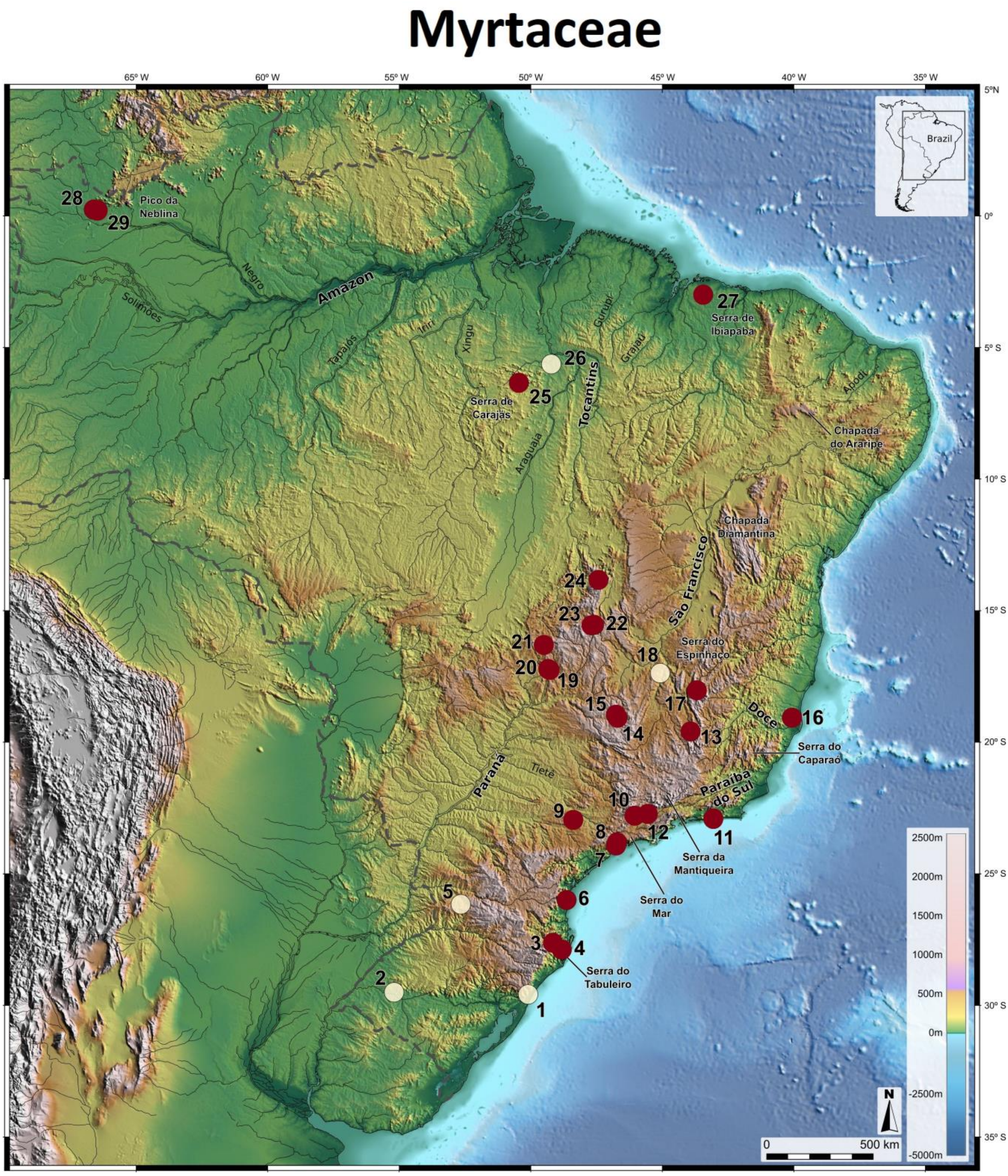

Supplementary Figure 5: Presence (red circles) and absence (clear circles) of Myrtaceae pollen in HS1 records of Brazil. Base layer: Shaded relief image of ETOPO1 Global DEM (continental area: shaded relief illumination from 060˚N, 30˚ above horizon, 40 times vertical exaggeration; oceanic area: illumination from 060˚N, 20˚ above horizon, 5 times vertical exaggeration).

Arecaceae

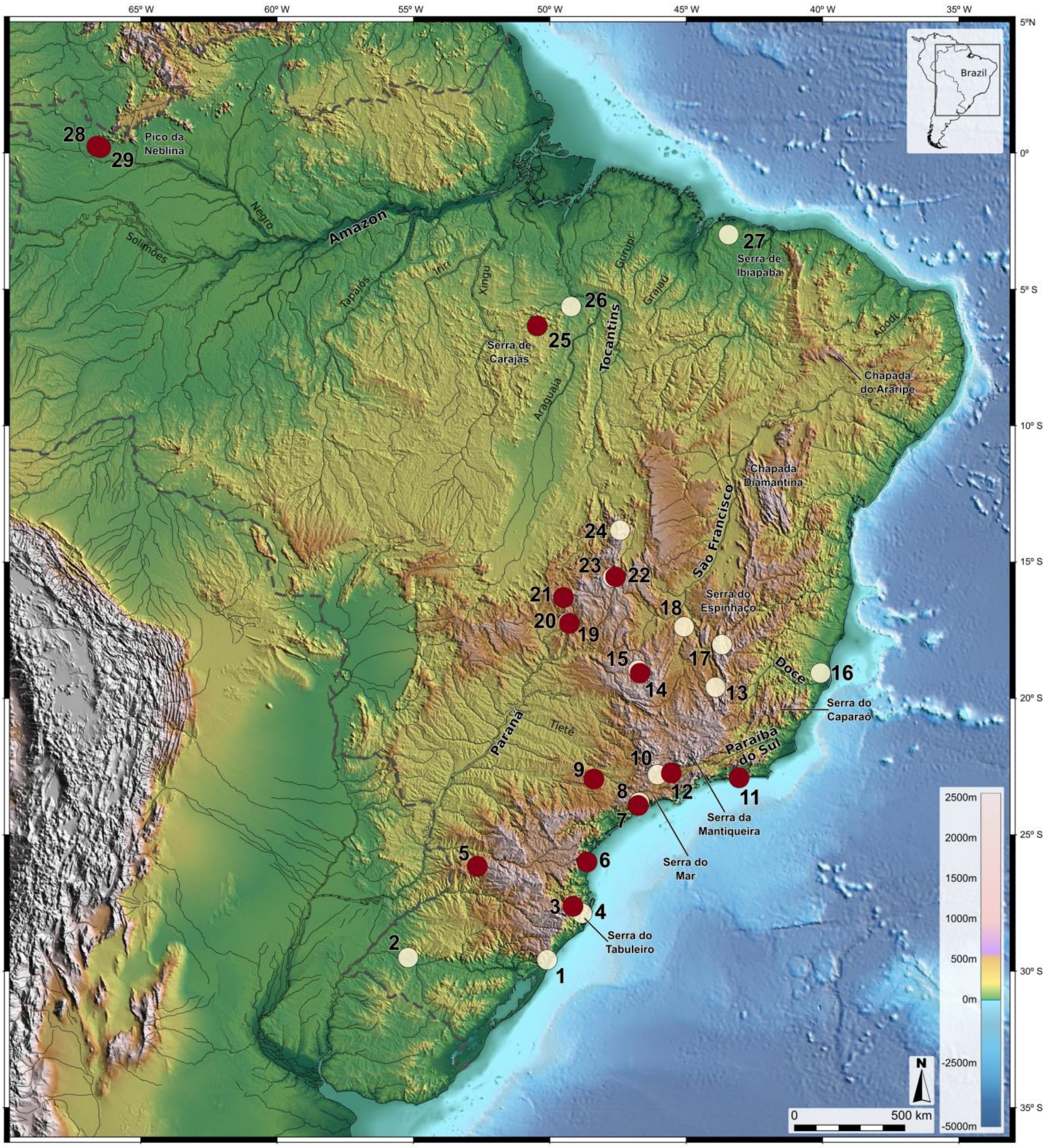

Supplementary Figure 6: Presence (red circles) and absence (clear circles) of Arecaceae pollen in HS1 records of Brazil. Base layer: Shaded relief image of ETOPO1 Global DEM (continental area: shaded relief illumination from 060˚N, 30˚ above horizon, 40 times vertical exaggeration; oceanic area: illumination from 060˚N, 20˚ above horizon, 5 times vertical exaggeration).

Ericaceae

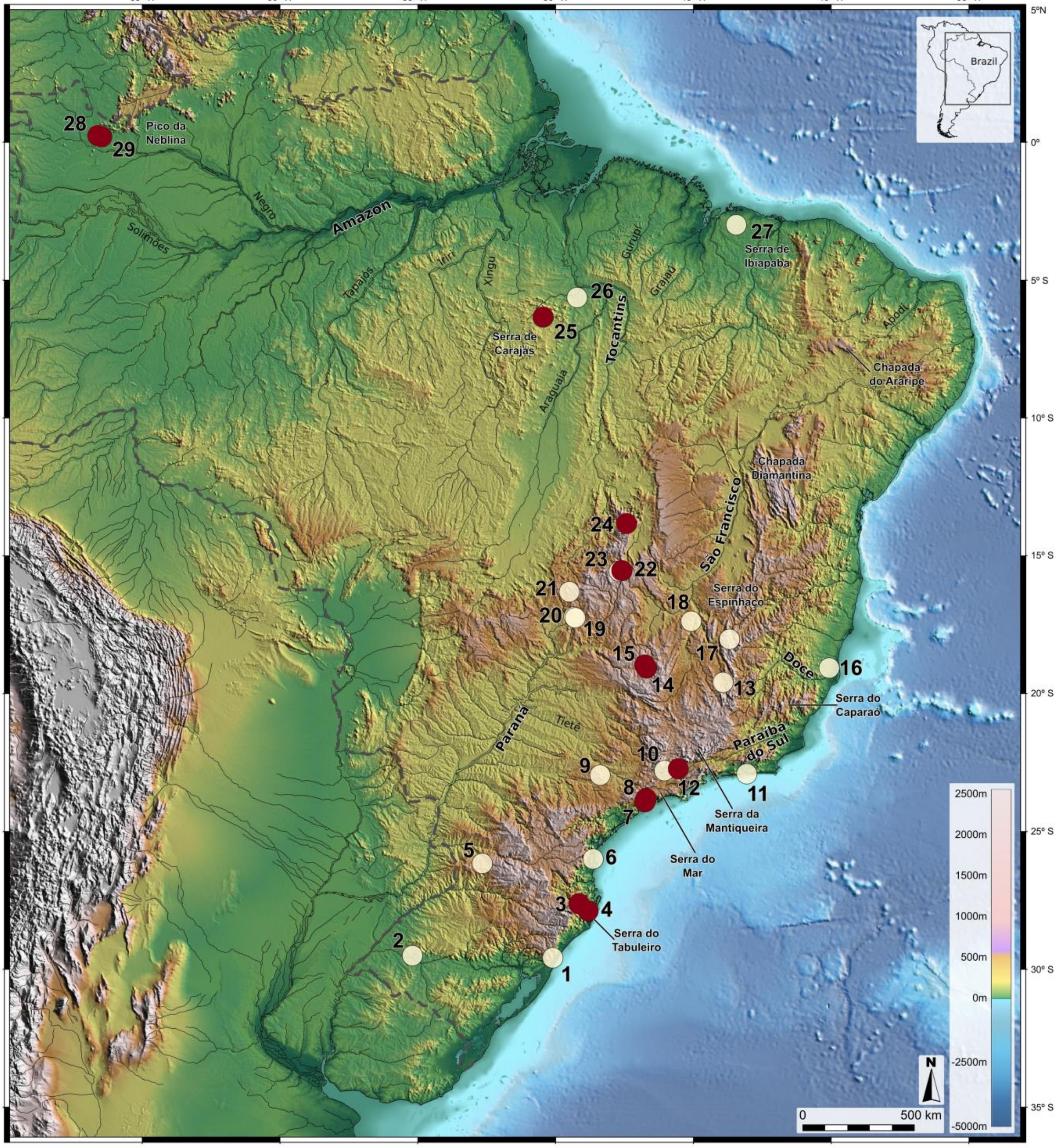

Supplementary Figure 7: Presence (red circles) and absence (clear circles) of Ericaceae pollen in HS1 records of Brazil. Base layer: Shaded relief image of ETOPO1 Global DEM (continental area: shaded relief illumination from 060˚N, 30˚ above horizon, 40 times vertical exaggeration; oceanic area: illumination from 060˚N, 20˚ above horizon, 5 times vertical exaggeration).

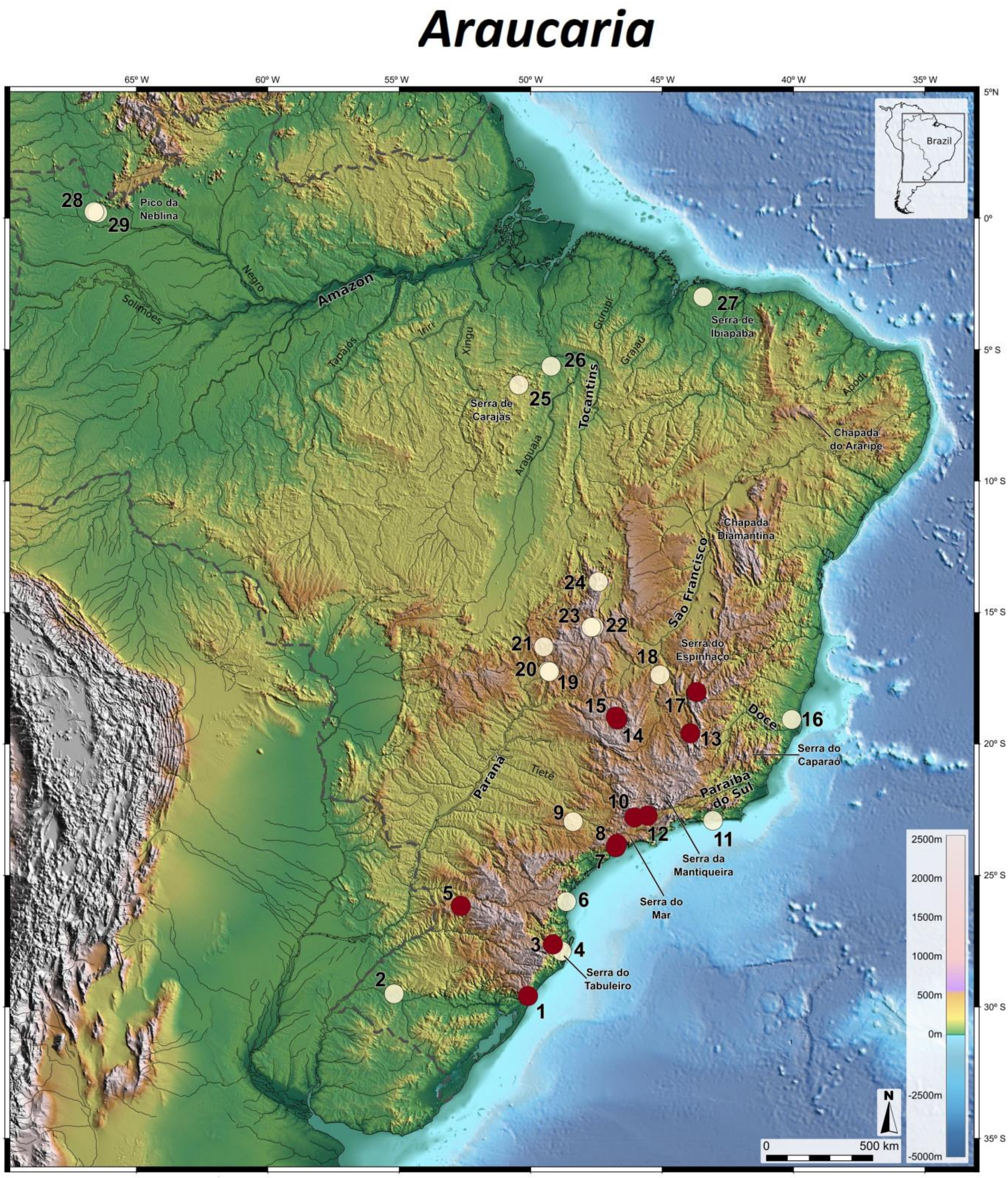

Supplementary Figure 8: Presence (red circles) and absence (clear circles) of *Araucaria* pollen in HS1 records of Brazil. Base layer: Shaded relief image of ETOPO1 Global DEM (continental area: shaded relief illumination from 060˚N, 30˚ above horizon, 40 times vertical exaggeration; oceanic area: illumination from 060˚N, 20˚ above horizon, 5 times vertical exaggeration).

Drimys

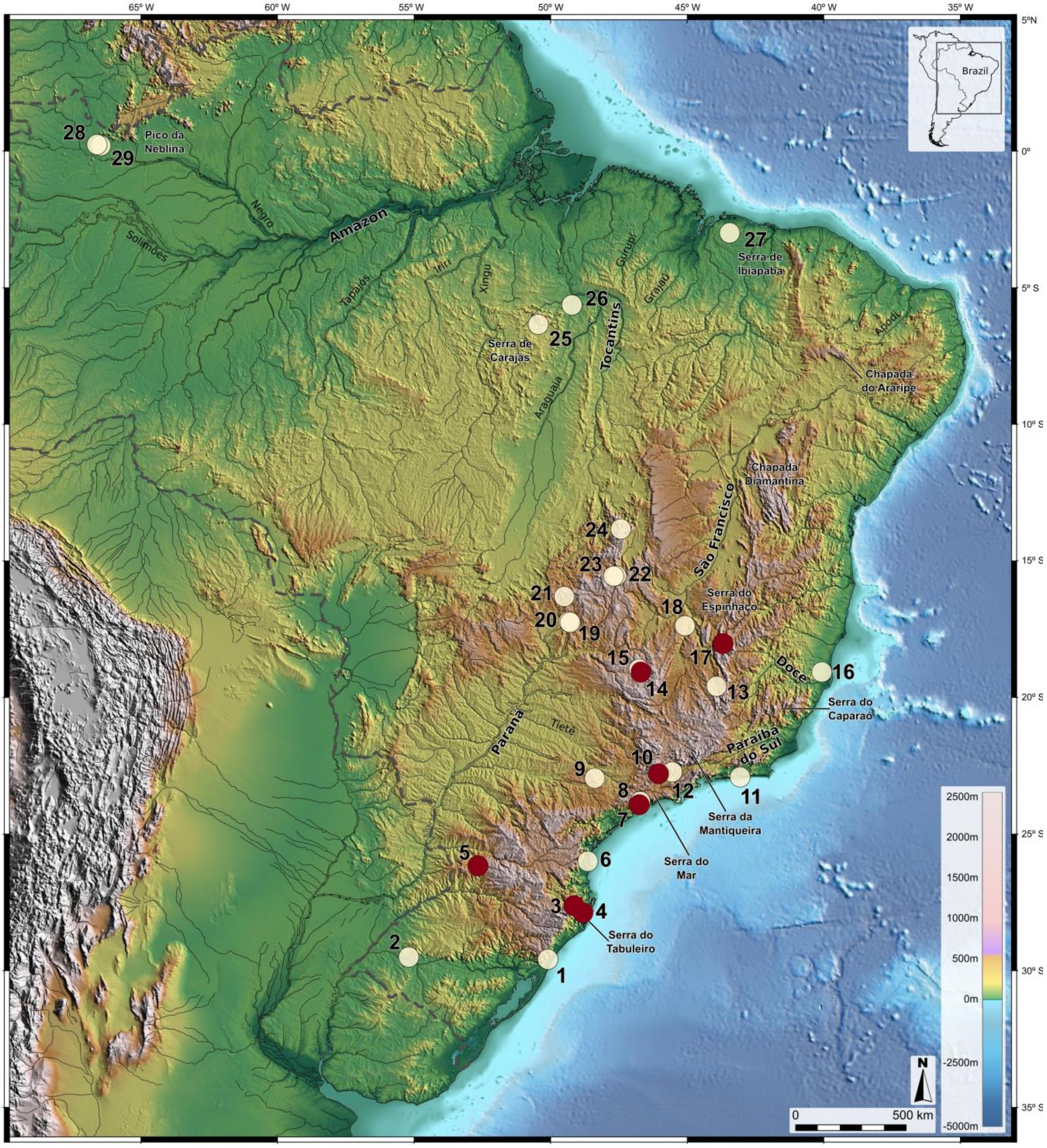

Supplementary Figure 9: Presence (red circles) and absence (clear circles) of *Drimys* pollen in HS1 records of Brazil. Base layer: Shaded relief image of ETOPO1 Global DEM (continental area: shaded relief illumination from 060˚N, 30˚ above horizon, 40 times vertical exaggeration; oceanic area: illumination from 060˚N, 20˚ above horizon, 5 times vertical exaggeration).

Symplocos

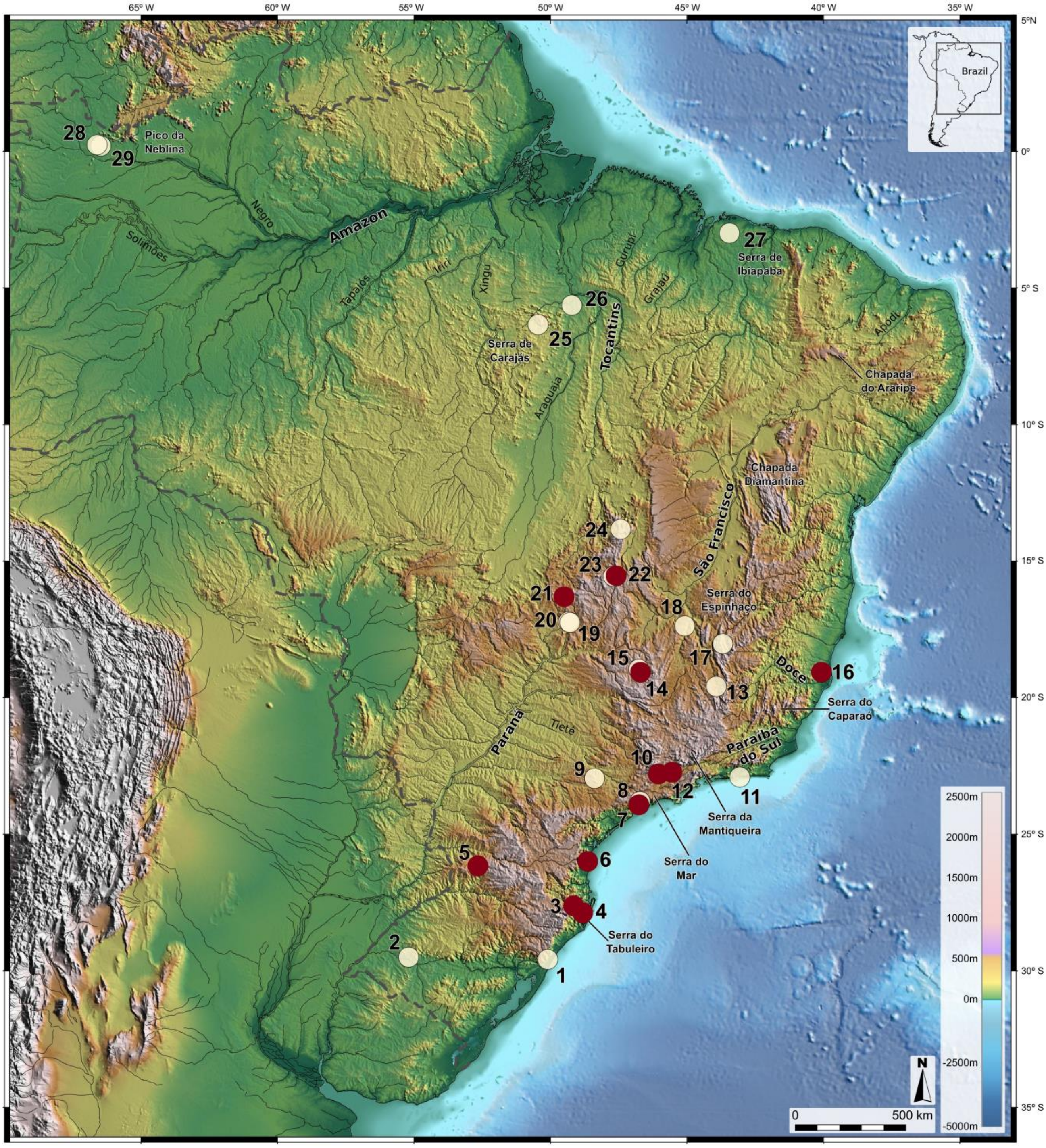

Supplementary Figure 10: Presence (red circles) and absence (clear circles) of *Symplocos* pollen in HS1 records of Brazil. Base layer: Shaded relief image of ETOPO1 Global DEM (continental area: shaded relief illumination from 060˚N, 30˚ above horizon, 40 times vertical exaggeration; oceanic area: illumination from 060˚N, 20˚ above horizon, 5 times vertical exaggeration).

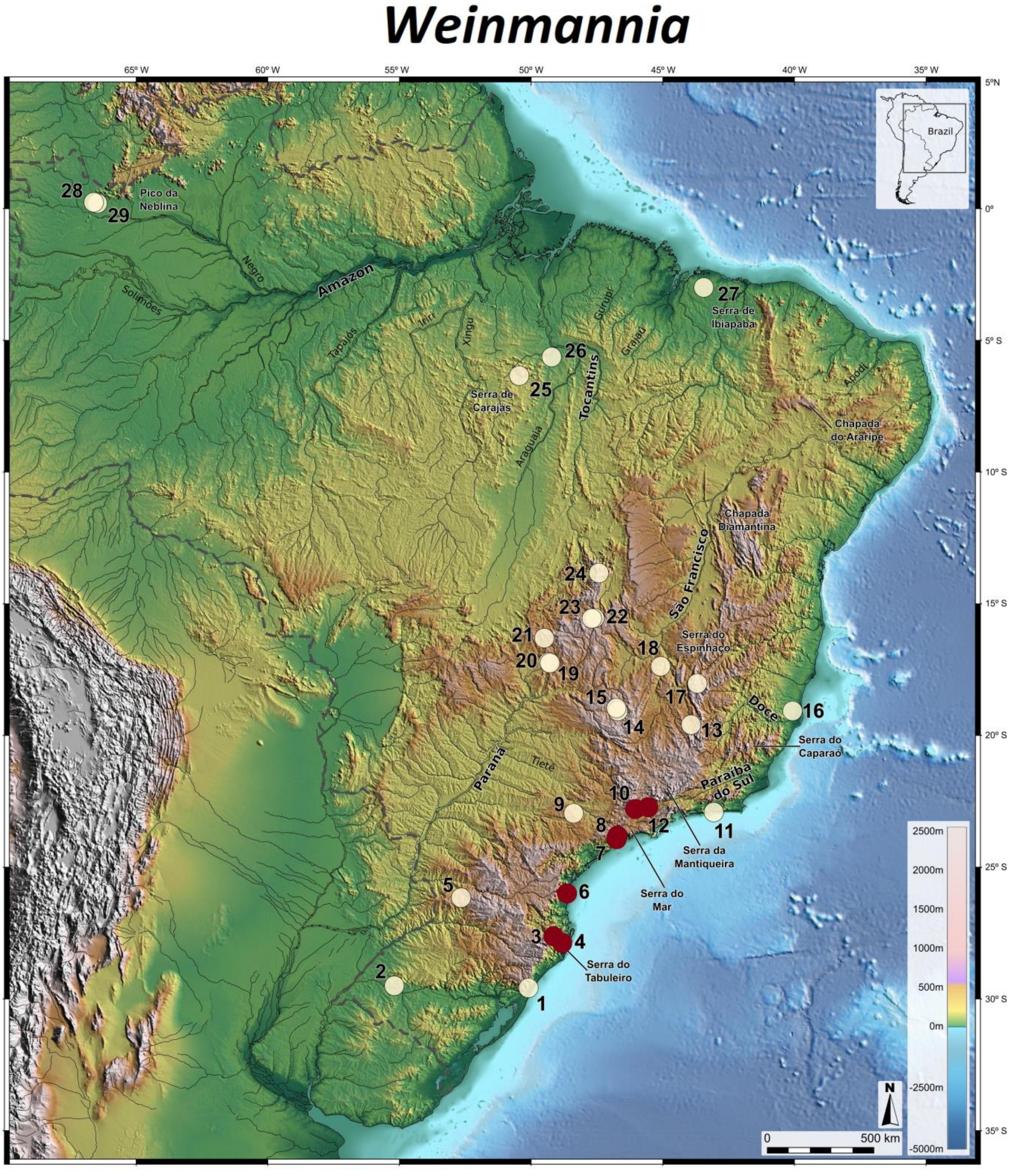

Supplementary Figure 11: Presence (red circles) and absence (clear circles) of *Weinmannia* pollen in HS1 records of Brazil. Base layer: Shaded relief image of ETOPO1 Global DEM (continental area: shaded relief illumination from 060˚N, 30˚ above horizon, 40 times vertical exaggeration; oceanic area: illumination from 060˚N, 20˚ above horizon, 5 times vertical exaggeration).
